# *In Situ* Cw ESR Study on Redox Behavior and Phase Heterogeneity in A-Site-Deficient Lanthanum Iron Manganite Perovskite Catalysts

**DOI:** 10.1021/acs.jpcc.4c08262

**Published:** 2025-04-07

**Authors:** Zohreh Asadi, Asghar Mohammadi, Thomas Ferdinand Winterstein, Ralf Feyerherm, Roham Talei, Nicolas Bonmassar, Wiebke Riedel, Simon Penner, Thomas Risse

**Affiliations:** a Institut für Chemie, Freie Universität Berlin, Arnimallee 22, Berlin 14195, Germany; b Institute of Physical Chemistry, University of Innsbruck, Innrain 52c, Innsbruck A-6020, Austria; c Institute Quantum Phenomena in Novel Materials, Helmholtz-Zentrum Berlin für Materialien und Energie GmbH, Hahn-Meitner-Platz 1, Berlin 14109, Germany; d Institute for Materials Science, University of Stuttgart, Heisenbergstr. 3, Stuttgart 70569, Germany

## Abstract

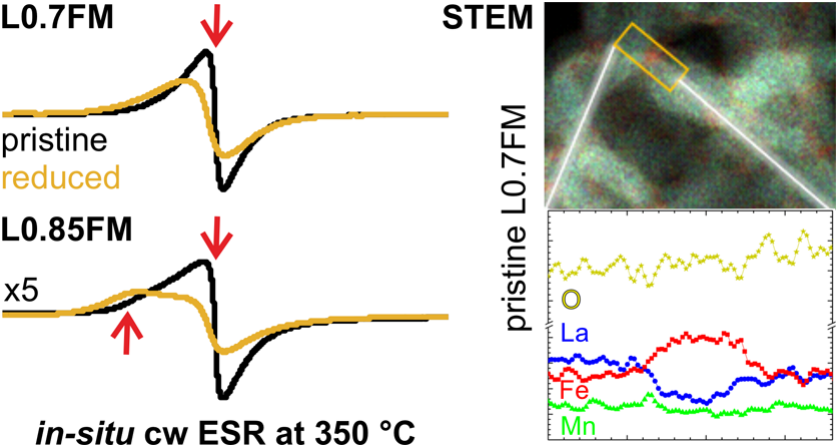

Temperature-dependent *in situ* continuous wave (cw) electron spin resonance (ESR) measurements are used for investigating changes in magnetization upon reduction and reoxidation in La-deficient La_*x*_Fe_0.7_Mn_0.3_O_3_ (*x* < 1) samples to advance the understanding of structural and chemical changes in these materials where A-site deficiency was shown to affect catalytic performance. For these defect-rich mixed perovskites with ferromagnetic ordering, the magnetic properties as characterized by *in situ* cw ESR spectroscopy are sensitive probes for structural changes in these materials. To this end, the ESR spectra observed in a redox cycle in H_2_- and O_2_-containing atmospheres not only show structural changes that were not observed by XRD but also provide evidence for heterogeneity in the magnetic phases, which notably depends on the La deficiency of the samples. This not only demonstrates the potential of such investigations for obtaining information complementary to other methods but also emphasizes the sensitivity of magnetic properties as probed by ESR to elucidate structural and chemical changes in such complex perovskite materials. While the XRD results lack indication for the presence of structural heterogeneity, STEM measurements provide evidence for a compositional heterogeneity between the grains but not for the presence of an additional magnetic phase, as observed by ESR for one of the samples. Importantly, the different magnetic phases exhibit distinct responses to reducing and (re)oxidizing atmospheres indicating for the sample with a lower La deficiency a facilitated reaction under reducing conditions at low temperatures but an overall higher structural stability. Both effects are expected to affect the reactivity in the redox reactions. Thus, these results provide new and complementary insights that can enhance the understanding of the effect of A-site deficiency in perovskite materials in redox reactions considered to be important for the catalytic activity of these systems.

## Introduction

Perovskites have attracted much attention for a range of applications including photovoltaic solar cells, fuel cells, batteries, and (photo/electro-)catalysts.^1−4^ By adjusting their composition, their optoelectronic, magnetic, and chemical properties can be tuned and, thus, optimized for a variety of applications. Namely, perovskites have been successfully used for a number of catalytic reactions.^4−7^ In this respect, promising results were obtained for the reduction of NO with CO important in three-way catalysts (TWC), which are used to remove harmful gases, such as NO_*x*_, CO, or unburnt hydrocarbons, from the exhaust of, e.g., automotive vehicles.^7−9^ Typically, these catalysts contain Pd or Pt for oxidation of CO and hydrocarbons as well as Rh for reduction of NO_*x*_, so that alternatives lowering the content of these expensive materials are economically very attractive.^10,11^

Different ABO_3_-type perovskites have been studied for this reaction.^12−15^ Often, materials with La occupying the A-sites were used, although partial substitution in the A-site with other metals, such as Sr, was also reported, whereas a variety of metals have been investigated for the B-site including Mn, Fe, Cu, or Co, as well as nonstoichiometric compositions of such perovskites.^12−15^ For mixed La(Fe,Mn)O_3_ perovskite catalysts (LFM) that show a high thermal stability and catalyst lifetime, a Mars–van Krevelen-type of reaction mechanism was suggested, and Mn^3+^/Mn^4+^ and Fe^3+^/Fe^4+^ redox couples were proposed to critically affect the catalytic activity.^13,16−18^ Accordingly, a deficiency of La in the A-site, which results in an increase in the average oxidation state of the B-site from nominally 3+, was found to improve the catalytic performance at low temperatures as compared to stoichiometric LaFe_0.7_Mn_0.3_O_3_.^19^ Comparing different degrees of La deficiency demonstrated a lower catalytic performance for “overdoped” La_0.7_Fe_0.7_Mn_0.3_O_3_ (L0.7FM) compared to La_0.85_Fe_0.7_Mn_0.3_O_3_ (L0.85FM) above temperatures of approximately 200 °C indicative for an optimum in La deficiency.^19^ Variation of the La deficiency may not only affect the average oxidation state of Mn and Fe in the B-site but may also induce oxygen vacancies, alter the compositions of the surface and bulk, or change the tendency for exsolution of Fe or Mn under reaction conditions.^5,19^

To understand which of these processes occur upon changing the La deficiency and thus affect the catalytic performance of this complex system, a variety of different analytic methods have been employed studying the catalysts not only *ex situ* but also *in situ* to provide insights into the differences under the relevant reaction conditions.^19^ In particular, *in situ* XPS and XRD measurements were used to evidence that the reaction of NO with CO has a net-reducing effect, which is significantly more pronounced for the “overdoped” L0.7FM sample and is associated with a lower degree of La enrichment at the surface, as compared to the less La-deficient materials. In combination with H_2_ and O_2_ uptake measurements, it was proposed that La deficiency facilitates oxygen diffusion out of the lattice but may also destabilize the material structurally resulting in phase heterogeneity under reductive conditions, especially when the optimum A-site deficiency is exceeded.^19^ The phase heterogeneity was, however, only evidenced by XRD at high temperatures, while differences in reactivity were detected already at lower temperatures.^19^

Comparable perovskites were often found to exhibit collective magnetic ordering including ferro-, ferri-, and antiferromagnetic behavior depending on the composition and resulting magnetic interactions.^20−23^ The intimate correlation between structure/composition and magnetic properties renders the latter a sensitive probe for the characterization of potential changes in the material. In line with expectations for samples with ferromagnetically coupled spins, prior *ex situ* ESR spectra of the La-deficient LFM samples^19^ studied here are characterized by broad signals due to ferromagnetically coupled spins lacking sharp signals characteristic of isolated paramagnetic species. Similar ESR results were reported for comparable perovskite samples.^5,6,15^ Oxygen-mediated superexchange interactions of Mn^3+^–O^2–^–Mn^4+^ pairs as well as double exchange interaction between Fe and Mn ions and defects affecting these interactions have been proposed for contributing to the magnetic properties in La_1–*x*_Mn(Fe)O_3_ perovskite samples.^6,21−25^ For LaMnO_3_, reaction-induced changes, such as defect formation, were also reported to affect the ESR signals.^15,26^ Hence, it is expected that the resulting magnetic properties for the LFM samples investigated here will depend on the type, concentration, and distribution of defect, as shown for similar perovskite samples.^6,21−25^ Therefore, *in situ* ESR spectroscopy is expected to provide complementary information as compared to previously reported *in situ* XPS and XRD measurements for these catalytic systems,^19^ as ESR spectroscopy does not require crystalline material and was shown to be able to probe changes in bulk as well as on the surface.^27^ Hence, it can yield information about disordered or defect-rich phases that may escape XRD detection.

In this work, temperature-dependent *in situ* cw ESR measurements were used to follow changes in (ferromagnetic) La-deficient LFM samples upon reduction in a H_2_-containing atmosphere and reoxidation in an O_2_-containing atmosphere. The L0.7FM and L0.85FM samples were chosen to allow for comparison with previous results from H_2_ and O_2_ uptake measurements and *in situ* XRD measurements for heating in H_2_ and (subsequently) O_2_, which indicated that A-site defects affect oxygen diffusion but also the structural stability of the material.^19^ Both of these measurements showed significant differences for the samples with varying degrees of La deficiency that were connected to reactivity differences observed in NO reduction with CO for temperatures above approximately 200 °C in the two samples. From a detailed analysis of the *in situ* ESR results, which requires to establish a suitable reference for measurements at various temperatures,^21,25^ it is possible to show that the degree of La deficiency has a direct impact on the homogeneity of the samples as well as their structural stability and response to different chemical environments, thus providing new insights, which can help to understand the differences in the catalytic properties. Thereby, these results may also contribute to future rational improvements of this class of materials.

## Experimental Section

Two lanthanum iron manganite (LFM) perovskite samples with different La deficiencies were synthesized employing a sol–gel approach, which has previously been described in detail.^19^ In short, the concentrations of the metal nitrate precursors were chosen to achieve nominal compositions of La_0.85_Fe_0.7_Mn_0.3_O_3_ (L0.85FM) and La_0.7_Fe_0.7_Mn_0.3_O_3_ (L0.7FM). The produced gel was calcined at 700 °C for 5 h. The pristine oxide samples obtained after calcination have been previously characterized in detail.^19^

*In situ* cw ESR measurements were conducted using a Bruker EMXplus equipped with a high-temperature resonator (Bruker, ER 4114 HT, TE011 mode), a specially designed quartz plug flow reactor, and a custom-built gas inlet system that have been described in detail before.^28^ For the measurements at different temperatures and in different gas atmospheres, the catalysts were placed in a quartz plug flow reactor with an inner diameter of 1 mm and a catalyst bed length of approximately 25 mm with the employed catalyst mass ranging from 8 to 13 mg.

For comparison with prior H_2_ and O_2_ uptake experiments,^19^ the samples used in the corresponding *in situ* cw ESR experiments were preoxidized directly before by heating in pure O_2_. In detail, the sample was heated to 430 °C (heating rate 10 °C min^–1^), kept there for 60 min, and subsequently cooled to room temperature (RT) in a constant oxygen gas flow (*p*(O_2_) = 1 bar, flow rate 1 mL min^–1^). In all other *in situ* cw ESR measurements, pristine samples were placed in the reactor and investigated without additional treatment.

For the *in situ* cw ESR measurements in different gas atmospheres, the samples were heated from RT to 400 °C with a heating rate of 6 °C min^–1^ followed by cooling to RT with a cooling rate of 10 °C min^–1^. To obtain the *in situ* cw ESR spectra at well-defined, constant temperatures, dwell times of 15 min were implemented at RT, 100 °C, 200 °C, 250 °C, 300 °C, 350 °C, and 400 °C in both heating and cooling steps. *In situ* measurements were conducted in pure N_2_, O_2_-containing (10 vol % O_2_ in N_2_), and H_2_-containing (10 vol % H_2_ in N_2_) atmospheres with the O_2_ and H_2_ contents matching those applied in previous H_2_ and O_2_ uptake measurements.^19^ The gas flow rate and pressure were set to 1 mL min^–1^ and 1 bar, respectively, for measurements to be compared to prior H_2_ and O_2_ uptake results, while the gas flow rate and pressure were adjusted to 0.66 mL min^–1^ and 1 bar, respectively, in all other *in situ* cw ESR measurements.

The temperature during the experiments was measured using a K-type thermocouple connected to the inlet of the reactor and in contact with the quartz wool holding the catalyst powder in place. As positioning of the thermocouple inside the catalyst bed is not possible due to the perturbation of the microwave resonator, the temperature of the catalyst bed was calibrated by an independent experiment. For this, an additional K-type thermocouple was placed inside the catalyst bed, and the temperature measured by both thermocouples was monitored during a stepwise increase in temperature up to 430 °C in a N_2_ flow rate of 1 mL min^–1^. The resulting calibration curve was used to give the temperature of the catalyst from the temperatures measured in front of the catalyst bed.

The cw ESR spectra were all recorded at X-band microwave (mw) frequencies (approximately 9.15 GHz) with a mw power of 2.00 mW applying a modulation frequency of 100 kHz and modulation amplitudes of 0.2 or 0.3 mT. The obtained spectra were normalized to account for intensity differences due to, e.g., the modulation amplitude, sample mass, or positioning of the catalyst in the resonator. The normalization was based on the RT measurements of the pristine samples, i.e., before any additional temperature treatments. The effective *g*-value *g*_eff_ was determined from the intersection of the measured cw ESR signals with a linear baseline and subsequently calculated by *h*ν = β_e_*Bg*_eff_ with the Planck constant *h*, the mw frequency ν, the Bohr magneton β_e_, and the magnetic field *B*.

*Ex situ* X-ray diffraction (XRD) measurements were performed by using a PANalytical Empyrean diffractometer equipped with a PIXcel^1D^ detector. The XRD data were collected in a Bragg–Brentano (BB) geometry utilizing Cu Kα_1_ radiation. Structure analysis was performed based on the references from the Inorganic Crystal Structure Database (ICSD) using TOPAS software (Bruker).

Scanning transmission electron microscopy (STEM) measurements were performed on a probe-corrected FEI Spectra300. The specimens were prepared by drop-casting a suspension consisting of 2-propanol and perovskite particles on a lacey carbon grid. We utilized high-angle annular dark-field (HAADF) imaging and energy-dispersive X-ray (EDX) spectroscopy (specimen-tilt independent Super-X EDS system) to characterize the morphology and the chemical composition of the perovskites.

## Results and Discussion

### Effect of Temperature on ESR Signal

**Figure 1:**
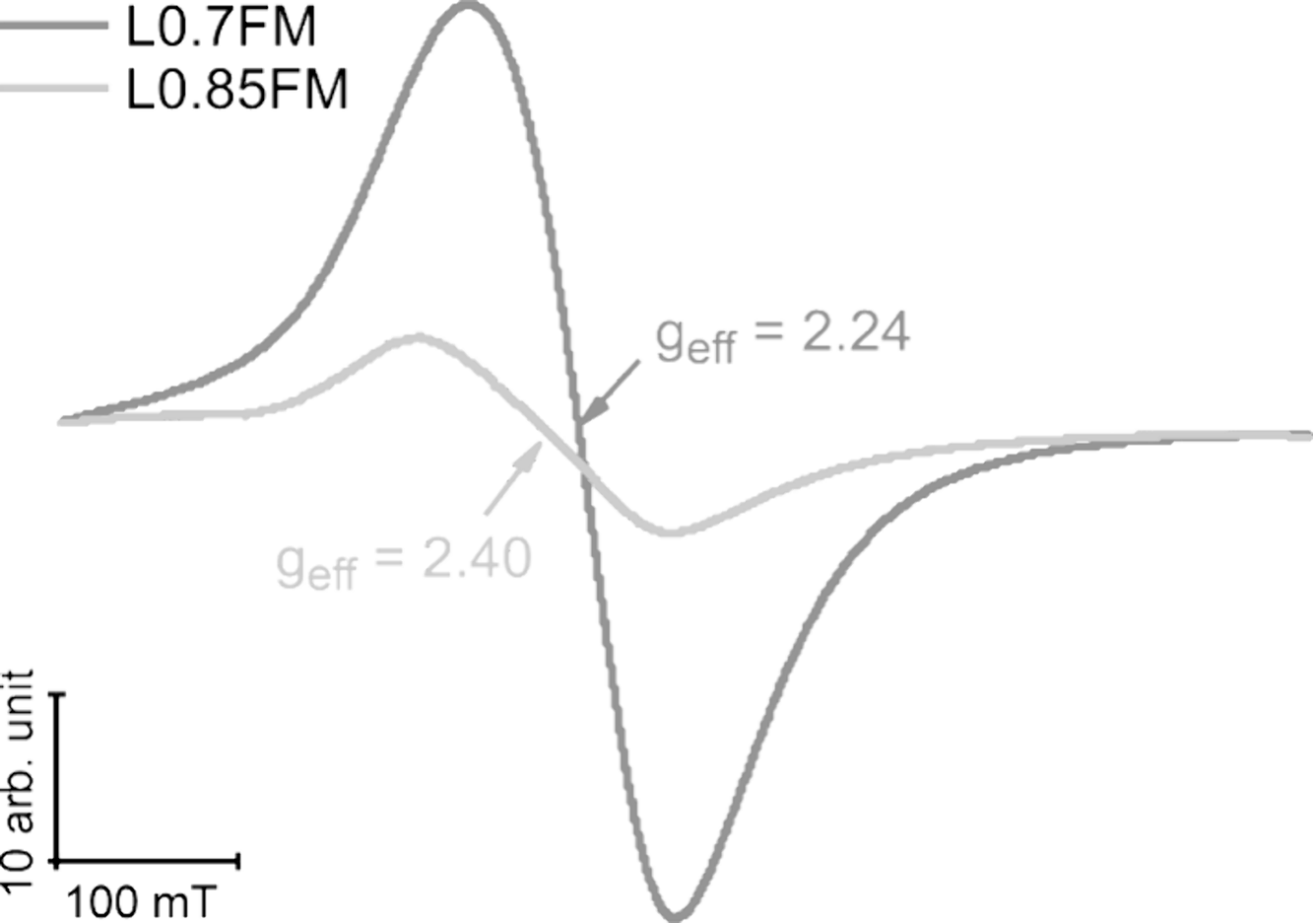
Cw ESR spectra of pristine L0.7FM (dark gray) and L0.85FM (light gray) obtained at RT.

Figure 1 shows the cw ESR signals of the pristine L0.7FM and L0.85FM samples measured at room temperature. As previously reported, both samples do not show any sharp signals but a broad signal (Δ*B*_pp_ > 100 mT).^19^ The absence of sharp signals characteristic of isolated species and the observation of broad signals are consistent with ferromagnetically coupled spin systems. In agreement, field-dependent magnetization measurements demonstrate at a low temperature (4 K) a magnetic field hysteresis expected for ferromagnetic order, while room temperature measurements suggest superparamagnetic behavior of the ferromagnetic materials expected for nanostructured materials (see also Figure S1). Temperature-dependent magnetization measurements indicate a Curie temperature >130 °C (limit of the device used in the magnetization measurements; see also Figure S1). The L0.7FM sample exhibits a significantly higher intensity (factor of 4) and a lower effective *g*-value *g*_eff_ than that of the L0.85FM sample. The intensity increase upon increasing La deficiency is consistent with previous measurements on nonstoichiometric LaMnO_3_.^5,6,15^ Changes in the ESR signals (or magnetization) of Mn-containing perovskite samples have been suggested to stem from oxygen-mediated superexchange interactions of Mn^3+^–O^2–^–Mn^4+^ pairs, and A-site deficiency was connected to changes in the ratio of Mn^3+^ and Mn^4+^.^6,21,22,24^ Yet, also double exchange interaction between Fe and Mn ions is among other proposed mechanisms of magnetic interactions in La_1–*x*_Mn(Fe)O_3_ perovskite samples.^25^ While a quantitative interpretation of the ESR signals in terms of speciation of the different defects expected to contribute to the signal is challenging, ESR spectra depend sensitively on the nature and distribution of defects, including those associated with La deficiency, allowing for valuable qualitative insights.

**Figure 2:**
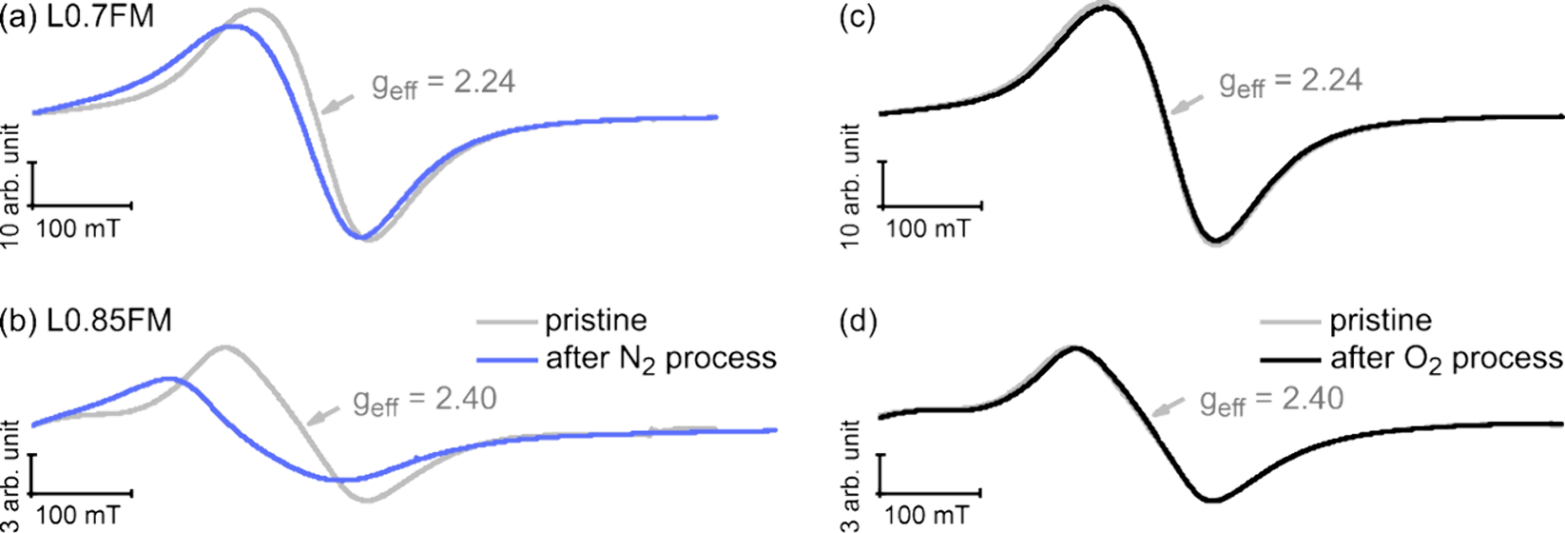
Cw ESR spectra obtained at RT of L0.7FM (a, c) and L0.85FM (b, d) before (i.e., pristine, gray traces) and after heating to 400 °C in pure N_2_ (blue traces, a and b) and in an O_2_-containing atmosphere (10 vol % O_2_ in N_2_, black traces, c and d).

As ESR signals of such ferromagnetic samples, which exhibit superparamagnetic behavior, typically change with temperature in shape and intensity, even in the absence of any chemical changes in the material (following not just a simple Curie-type behavior, as expected for isolated paramagnetic spin species),^21,25^ first, a proper set of reference spectra is required whose temperature-dependent spectra are solely due to the changes in spin physics. These reference measurements are needed to disentangle spectral changes due to temperature-induced changes in the magnetization dynamics from changes of the magnetization induced by chemical (or structural) changes in the material. To avoid chemical changes, an atmosphere preventing changes in the chemical composition is required for these reference measurements. Typically, such measurements are conducted in inert gases, such as N_2_ or noble gases. Accordingly, the first set of temperature-dependent measurements of the pristine L0.7FM and L0.85FM samples was conducted in pure N_2_ by heating the samples stepwise up to 400 °C and cooling to RT, as described in the Experimental Section. The cw ESR spectra measured at RT in N_2_ before and after heating in pure N_2_ to 400 °C are displayed in Figure 2a,b for L0.7FM and L0.85FM, respectively. After heating in pure N_2_, clearly, changes in the RT spectra are detected for both samples including changes in the effective *g*-value, line width, and intensity. Similarly, the spectra taken at elevated temperatures during heating and cooling differ even for the same temperature (Figure S2). This implies that heating in N_2_ to 400 °C causes irreversible structural or chemical changes. In agreement, *ex situ* XRD measurements of the samples before and after heating in pure N_2_ to 400 °C show a notable shift of the reflexes of the perovskite phase to lower angles and accordingly an increase in the lattice parameters and unit cell volume after heating in pure N_2_ (see Table 1 and Figure S3). Comparing the two La-deficient samples, the change in both the ESR signals and the unit cell parameters determined from XRD is more pronounced for the L0.85FM sample, which also demonstrates the dependence of these changes on the degree of La deficiency. These results clearly show that the spectra obtained for heating in pure N_2_ cannot serve as a reference to discriminate the effect of spin dynamics from that of chemical or structural changes.

**Table 1:** Results of *Ex Situ* XRD Measurements of Pristine LFM Samples as well as after Heating in Pure N_2_, in an O_2_-Containing Atmosphere, and after Reoxidation by Heating in an O_2_-Containing Atmosphere of Samples Reduced by Heating in a H_2_-Containing Atmosphere

	**cell parameters [Å]**				
**sample processing**	*a*	*b*	*c*	**cell volume [Å**^**3**^**]**	**crystallite size [nm]**
L0.7FM					
pristine	5.505	5.554	7.790	238.18	22.8
O_2_ heated	5.506	5.554	7.790	238.20	22.7
N_2_ heated	5.519	5.563	7.802	239.54	23.1
reoxidized	5.513	5.554	7.794	238.64	24.6
L0.85FM					
pristine	5.512	5.555	7.797	238.71	25.5
O_2_ heated	5.511	5.556	7.796	238.71	25.6
N_2_ heated	5.544	5.571	7.824	241.65	28.0
reoxidized	5.516	5.554	7.797	238.87	26.0

Whereas gases such as N_2_ or noble gases are traditionally considered “inert”, the low chemical potential of oxygen in the gas phase may allow for reduction of oxides at elevated temperatures. Thus, the changes observed by ESR and XRD upon heating in pure N_2_ may be connected to a reduction of the LFM samples, resulting in a change in their chemical composition. While changes in the La-deficient perovskite materials upon heating in pure N_2_ (or, e.g., noble gases) were not addressed in the previous study^19^ to support this hypothesis, it is expected that proper reference spectra should be taken in a more oxidizing atmosphere to prevent a thermal reduction of the system. To this end, the samples have been subjected to the same temperature treatment using an atmosphere containing 10 vol % O_2_ in N_2_.

The ESR spectra taken at RT before and after heating in the O_2_-containing atmosphere are displayed in Figures 2c and 2d for L0.7FM and L0.85FM, respectively. For these measurements, the spectra before and after the sample was heated to 400 °C do not exhibit significant changes. This also holds at elevated temperatures comparing spectra taken under these conditions at the same temperature during heating and cooling (see Figure S4). In agreement with the *in situ* ESR results, also *ex situ* XRD measurements of the samples before and after heating in the O_2_-containing atmosphere do not show significant changes, in contrast to the samples heated in pure N_2_ (see Table 1 and Figure S3). This supports the interpretation of a temperature-induced reduction of the samples upon heating in pure N_2_. It should be noted that the ESR spectra obtained at high temperatures for heating in the O_2_-containing atmosphere (10 vol % O_2_ in N_2_) do not differ notably from spectra obtained in pure O_2_ (see also Figure S5), indicating that the samples cannot be further oxidized even by increasing the chemical potential of oxygen significantly. In turn, possible oxygen vacancies present in these samples are stable over a fairly large range of oxygen chemical potentials.

The perfect reversibility of the spectra at RT before and after annealing in an O_2_-containing atmosphere renders the corresponding temperature series a suitable reference, as the observed changes of the ESR line shape and intensity must be attributed to temperature-induced changes of the spin dynamics. Please note that the results presented above attests to the point that a characterization of the temperature-dependent magnetic properties oftentimes reported in the literature by heating the samples in an “inert” atmosphere should be taken with caution in case that the reversibility of these properties prior to and after the temperature treatment is not shown explicitly.

### Thermal and Chemical Reduction

To investigate how the magnetization of the L0.7FM and L0.85FM samples changes upon chemical reduction, *in situ* ESR measurements were conducted for heating the samples to 400 °C in 10 vol % H_2_ in N_2_. The H_2_-containg atmosphere is expected to be more reducing than just pure N_2_, as H_2_ may add a reaction channel to remove lattice oxygen by water formation in addition to the temperature-induced oxygen evolution in a pure N_2_ atmosphere.

For these measurements, the pristine L0.7FM and L0.85FM samples were first preoxidized by heating to 430 °C in a pure oxygen flow. This procedure was adapted to allow for a more direct comparison with a previously reported uptake study using these samples despite the fact that ESR does not show significant changes after this treatment (see discussion above and Figure S6).^19^

**Figure 3:**
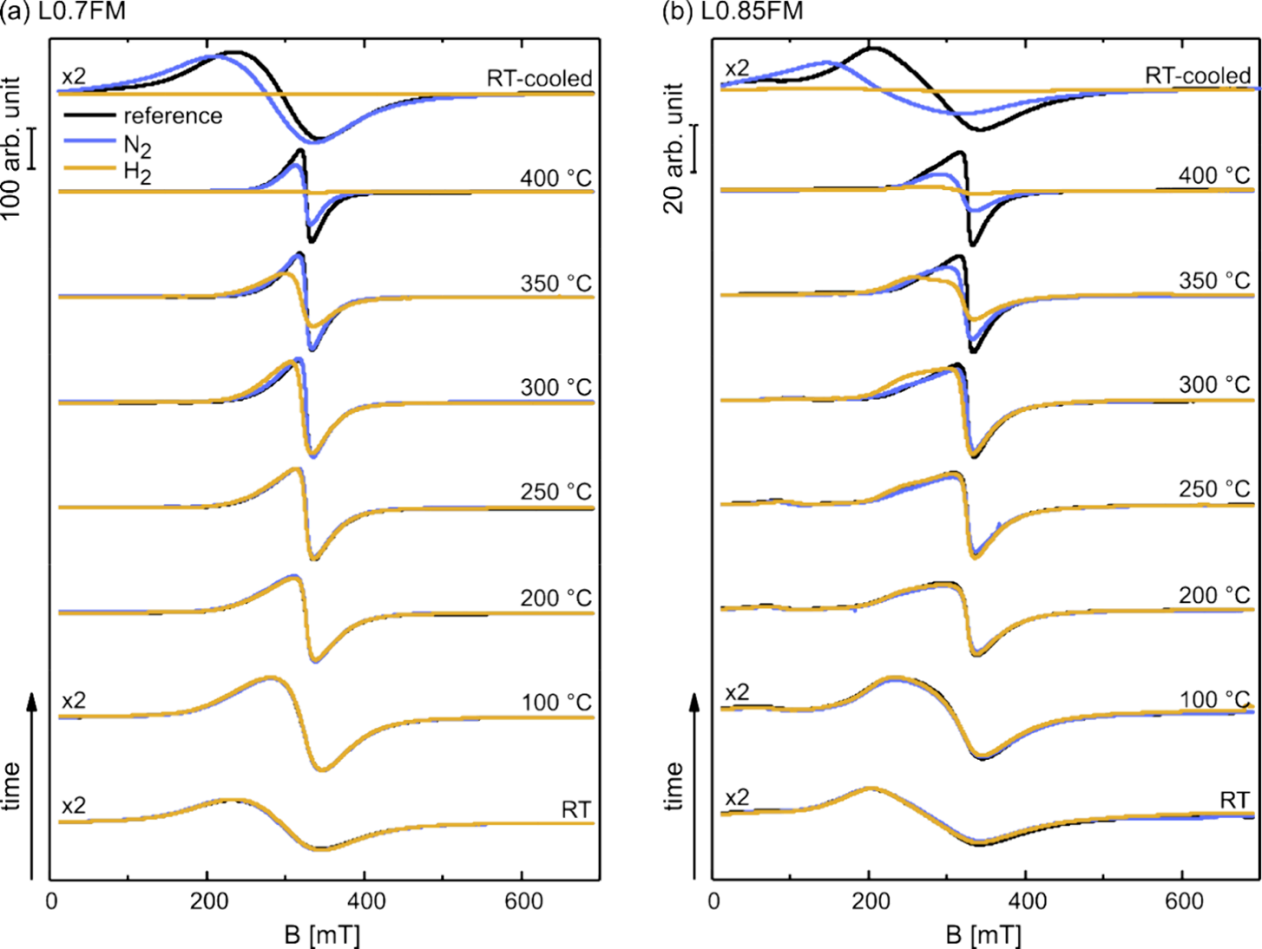
*In situ* cw ESR measurements of (a) L0.7FM and (b) L0.85FM obtained at different temperatures for stepwise heating in a H_2_-containing atmosphere (yellow traces) and in pure N_2_ (blue traces) displayed together with the reference measurements obtained by heating in an O_2_-containing atmosphere (black traces).

The *in situ* cw ESR spectra obtained for heating the preoxidized LFM samples in a H_2_-containing atmosphere (10 vol % H_2_ in N_2_; yellow traces) are displayed in Figure 3 for both L0.7FM and L0.85FM samples together with the corresponding reference spectra, i.e., for heating in 10 vol % O_2_ in N_2_ (black traces). Moreover, the *in situ* cw ESR spectra for heating in pure N_2_ are shown for comparison (blue traces). Heating in the H_2_-containing atmosphere results in changes of the ESR signals for both L0.7FM and L0.85FM, compared to the reference measurements. For L0.7FM heated in the H_2_-containing atmosphere, notable signal deviations from the reference are observed for temperatures ≥300 °C characterized by a clear shift in the effective *g*-value to higher values and a decrease in the signal amplitude and intensity (see also Figure S7). At 400 °C, the ESR signal nearly vanished. Cooling to RT does not change this: no significant ESR signal can be detected. Thus, the magnetic phase responsible for the ESR signal before heating in a H_2_-containing atmosphere is strongly altered by the reactions with hydrogen. The onset in ESR signal deviations from the reference spectra, when heating in a H_2_-containing atmosphere, coincides with the onset of a significant H_2_ uptake reported previously.^19^ Presumably, oxygen removal by reactions with hydrogen lowers superexchange interactions but may also lower, e.g., the Mn^4+^ content and thereby the magnetization of the sample resulting in the loss of ESR intensity.^15^ Note that the disappearance of the ESR signal at 400 °C coincides with a notable reduction in H_2_ uptake,^19^ suggesting that the reduction of the ferromagnetic perovskite phase resulting in a loss of its ESR signal is essentially completed at this temperature. Some H_2_ uptake was already observed at lower temperatures (≥170 °C)^19^ where the ESR measurements did not show any significant deviations for the L0.7FM sample from the reference measurements, indicating that the magnetic properties remain unaffected for the L0.7FM for the initial stages of hydrogen uptake.

The *in situ* ESR signals of L0.85FM also exhibit a shift in the effective *g*-value to higher values and a decrease in signal intensity at elevated temperatures upon heating in H_2_-containing atmospheres as compared to the reference spectra. While this behavior is qualitatively similar to the L0.7FM sample, first deviations from the reference measurements are already detected after heating in a H_2_-containing atmosphere at 200 °C. At that temperature, a shoulder at lower magnetic fields, which is also present in the reference spectrum, gains intensity and remains prominent up to 300 °C, the temperature above which the ESR signal intensity starts to decrease in comparison to the reference measurements. The shoulder at lower magnetic fields is also present in the reference measurements of L0.85FM at elevated temperatures, which is in marked contrast to the L0.7FM sample lacking an indication for such a spectral component under any of the applied conditions. The observation of a second feature at lower magnetic fields has been previously reported for Na-doped LaMnO_3_ with a high concentration of Mn vacancies and was attributed to a second magnetic phase.^21^ Similarly, additional features in the cw ESR spectra of LFM samples were previously detected, especially prominent for samples with low Fe contents and were attributed to magnetically different phases in these samples.^25^ Accordingly, the second feature at lower magnetic fields of the L0.85FM sample is assigned to an additional magnetic phase, which differs from that associated with the signal closer to *g*_eff_ ≈ 2. Please note that the signal at a higher magnetic field (*g*_eff_ ≈ 2) of the L0.85FM sample exhibits a similar temperature dependence as the signal observed for the L0.7FM discussed above, whereas the signal at a lower field shows a higher thermal stability, as seen from the spectrum taken at 350 °C. The latter observation supports the assignment of these two signals to two magnetically different phases of the sample. At 400 °C, the ESR signal of the L0.85FM sample nearly vanished and does not gain notable intensity upon cooling to RT, which is similar to the results obtained for the L0.7FM sample (see Figure S7). Please note that previous *in situ* XRD of samples reduced in a pure H_2_ atmosphere provides evidence for the exsolution of iron and the formation of a metallic iron phase.^19^ The lack of ESR signals after cooling down to low temperatures excludes this process for both samples, if reduction is done in an atmosphere containing only 10 vol% H_2_ in N_2_.

Along the same line discussed above, the changes of the ESR signal at high temperatures are interpreted as being due to a removal of lattice oxygen, finally resulting in a loss of ferromagnetic order due to reduced superexchange interactions and/or Mn^4+^ content. Alike the results of the L0.7FM sample, the strong decrease in ESR signal intensity at 400 °C also coincides for the L0.85FM sample with a notably slowed down H_2_ uptake that has been reported previously.^19^ This agrees with the suggested picture that the removal of lattice oxygen, which we associated with the loss of the ESR signals, is (nearly) complete at this temperature. Comparing the two samples, the relative deviations of signal intensity at 400 °C from the references are significantly larger for the L0.7FM sample. This is consistent with a previously reported overall higher H_2_ uptake at 400 °C for L0.7FM.^19^ The onset of the decrease in ESR intensity for heating in a H_2_-containing atmosphere also coincides for L0.85FM with an increased H_2_ uptake, as measured in a prior study.^19^ Please note that in this temperature range (>200 °C), where the ESR spectra of L0.85FM start to change in a H_2_-containing stream, also a higher reactivity for L0.85FM compared to L0.7FM in the NO reduction with CO was previously reported.^19^

**Figure 4:**
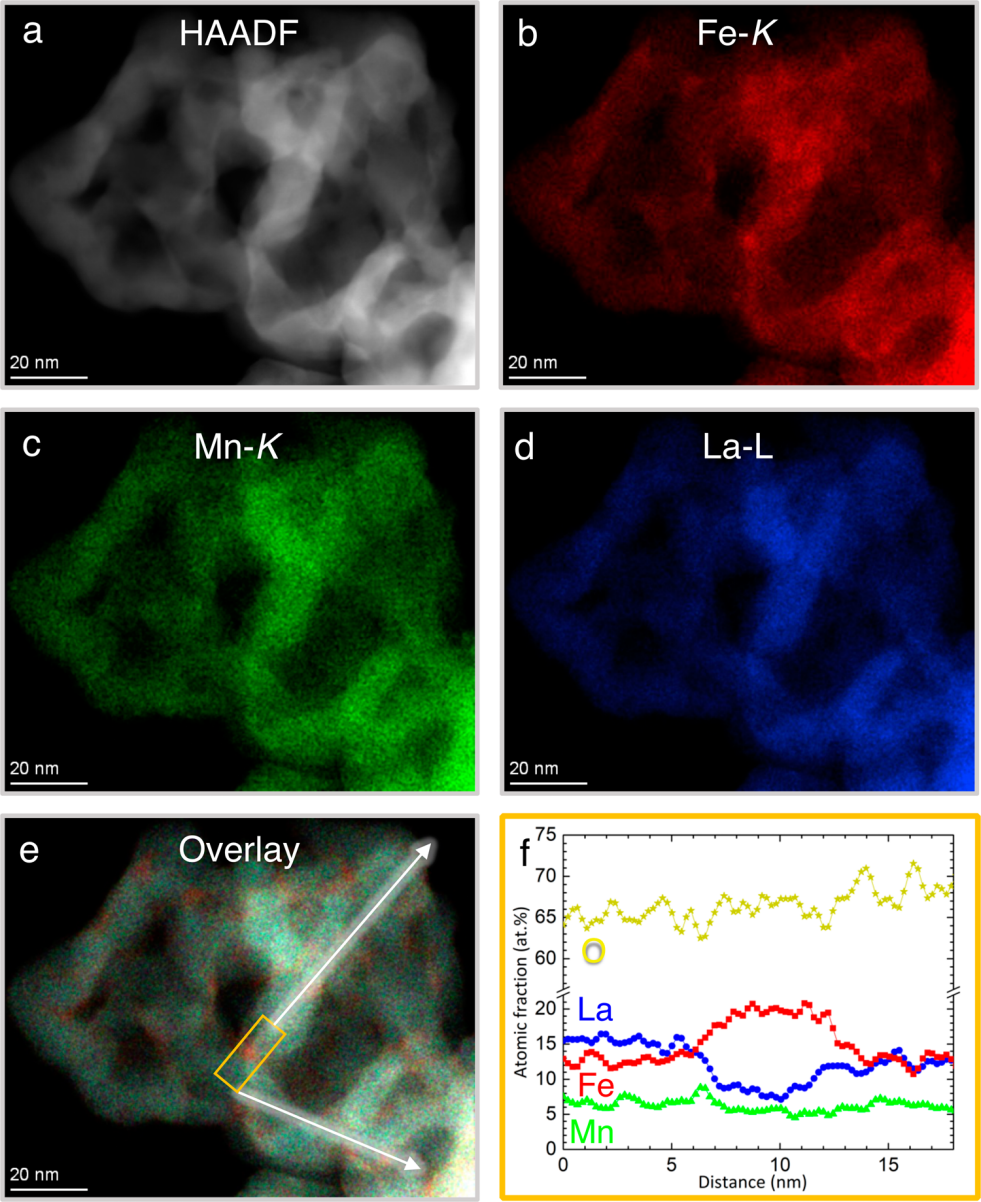
Nanoscale morphological and quantitative chemical analysis of L0.7FM using HAADF and EDX mapping. (a) HAADF overview image showing agglomerated perovskite nanoparticles. (b–d) Elemental mappings of Fe-K (red), Mn-K (green) and La-L (blue) showing a heterogeneous distribution of Fe; Mn appears homogeneous. (e) Overlay of Fe, Mn, and La signals visualizing the position of the Fe-rich areas, which are located at the interface between nanoparticles. (f) Elemental line profile highlighting the atomic fraction integrated at the position of the orange rectangle shown in panel e.

The different magnetic components being observed already for the pristine L0.85FM imply heterogeneity in this sample, while only one crystalline phase was found in the XRD of the pristine sample. As the different magnetic phases are also evidenced in the reference spectra, the pristine samples are expected to exhibit this heterogeneity. To further investigate the samples for potential heterogeneities in the material, STEM measurements were performed. In Figure 4, the STEM results are displayed for the pristine L0.7FM. The HAADF overview image in Figure 4a shows an agglomeration of nanoparticles with sizes ranging from 10 to 20 nm. Considering the EDX results (Figure 4b–e), we could identify Fe-rich areas at the interface between these nanoparticles could be identified. In some regions at the interface between nanoparticles, an enrichment in iron coinciding with a low La content is observed, as indicated in the elemental line profile in Figure 4f. These Fe-rich and La-depleted regions are more pronounced for L0.7FM than for L0.85FM (see also Figure S8), indicating that the heterogeneity depends on the degree of La deficiency. This also implies that the composition in the particles differs for the two samples. This should also affect the magnetic properties because the magnetic coupling between different cation pairs can be ferro-, ferri-, or antiferromagnetic depending on the type of cation, oxidation state, and the distribution in the perovskite, e.g., while Mn^4+^–O–Mn^3+^ ferromagnetic coupling is expected, antiferromagnetic coupling was suggested for Mn^3+^–O–Mn^3+^.^20−23,29^ Consequently, these compositional differences detected by STEM should affect the signals detected by cw ESR and may be connected to, e.g., the significantly higher intensity of the cw ESR signal around *g*_eff_ ≈ 2 in the L0.7FM sample. While the STEM measurements clearly demonstrate a local heterogeneity in the element distribution between two perovskite particles, the images provide no evidence for the presence of a second magnetic phase as seen by cw ESR for L0.85FM. It is expected that the inhomogeneous distribution within the grains will also affect the magnetic properties; however, a quantitative analysis of the ESR spectra is hampered by the complex superposition of lines expected for such a heterogeneous sample, which cannot be disentangled with the available information.

As noted before, these two magnetic phases respond very differently to a H_2_-containing atmosphere at low temperatures, despite similar H_2_ uptake up to 280 °C.^19^ H_2_ uptake was observed for both La-deficient samples at lower temperatures than for the stoichiometric LFM reference, indicating that defects induced by the La deficiency facilitate the interaction with H_2_.^19^ Due to the low temperatures, it is reasonable to assume that only processes with rather low activation barriers can occur, such as oxygen removal from the surface region rather than from the bulk. Previous XPS measurements of the pristine samples reported for the L0.85FM sample remnants of a La enrichment at the surface, which is more pronounced in stoichiometric LFM but is absent in the L0.7FM samples lacking also signals due to oxygen species connected with this La-enriched surface.^19^ For the L0.7FM sample, which exhibits only the signal at *g*_eff_ ≈ 2, no change in the cw ESR signal compared to the reference was detected for the low-temperature H_2_ uptake. Assuming that mainly the surface modified by the La deficiency is affected by the H_2_ uptake suggests in turn that the surface layer of L0.7FM, i.e., without the La enrichment and associated oxygen species, does not significantly contribute to the magnetization detected by cw ESR. A possible scenario is an antiferromagnetic coupling of the spins, which is not lifted or whose Neél temperature is not sufficiently lowered by the low-temperature H_2_ uptake.

While the signal at *g*_eff_ ≈ 2, which is the only one observed for L0.7FM, does not change at low temperatures compared to the reference, the shoulder at lower magnetic fields detected for L0.85FM starts to increase in intensity at a temperature close to the onset of H_2_ uptake^19^ and grows until 300 °C. The increase in the ESR signal between 200 and 300 °C is not in line with the simplifying picture that reduction by H_2_ results in a loss of lattice oxygen and in turn reduced magnetization of the material. This raises the question whether other chemical processes, such as hydrogen diffusion into the system, might be at play. While additional processes cannot be ruled out completely, it is interesting to note that the low-temperature signal also increases in intensity if the system is heated in pure N_2_ (see also Figure S9). Although the effect is much less pronounced, this result indicates that an increase in this ESR signal at lower magnetic fields is possible also in the absence of hydrogen, which shows that the latter is not mandatory for an increased ESR signal. This suggests that this magnetic phase may gain in magnetization by the removal of lattice oxygen. A possible scenario would be that the pristine phase has at least some (ferri- or) antiferromagnetically coupled spins and that a reduction of this phase lifts some of the antiferromagnetic coupling increasing the fraction of the spins with (ferri- or) ferromagnetic coupling and thus the magnetization detected by cw ESR. While antiferromagnetic ordering of the surface layer of the L0.7FM sample may cause the absence of a second signal in the cw ESR measurements for this sample, it is possible for the L0.85FM exhibiting a second cw ESR signal that the compositional differences in the surface layer induce a rather ferrimagnetic ordering allowing for detection by cw ESR. Differences in site occupancy due to a varying degree of La deficiency may be inferred from the STEM measurements evidencing regions with Fe enrichment that are less pronounced for a lower degree of La deficiency. Furthermore, previous XPS measurements showed for pristine L0.85FM remnants of a La enrichment and corresponding oxygen species at the surface, which were absent for L0.7FM.^19^ On the one hand, the magnetic properties for such a compositionally altered surface may respond differently upon reduction than those for a surface without the La enhancement and according oxygen species. On the other hand, the compositional change may also affect the reduction process itself. Previous *in situ* XPS investigations showed differences in the surface oxygen evolution during the net-reductive reaction of NO with CO for the two La-deficient LFM samples with the L0.85FM exhibiting a strong decrease in the surface oxygen content, which was attributed to changes in the oxygen mobility.^19^ A similar effect may contribute to the observed differences in cw ESR signals of the two La-deficient samples upon reduction in H_2_-containing atmospheres.

For heating the samples in pure N_2_, the ESR signal around *g*_eff_ ≈ 2 shows deviations from the reference only around 350 °C for L0.85FM and for L0.7FM even above 350 °C, which are higher temperatures as compared to the onset temperature of approximately 300 °C for heating in a H_2_-containing atmosphere. Hence, reactions with hydrogen facilitate the reduction of the samples already at lower temperatures as compared to mere thermally induced oxygen diffusion out of the lattice, as expected for heating in pure N_2_. The onset temperature for the deviations for heating in pure N_2_ from the references is somewhat lower for L0.85FM suggesting an easier thermal reduction of the sample with a lower La deficiency also leading to a larger deviation of the signal at 400 °C for this sample, as compared to L0.7FM. However, the deviations for heating in pure N_2_ are overall smaller for both samples, as compared to heating in a H_2_-containing atmosphere, as seen by the fact that both samples exhibit a significant ESR signal after heating to 400 °C in pure N_2_, while the ESR signal is quenched if the sample is heated in a H_2_-containing atmosphere at the same temperature.

### Reoxidation of Chemically Reduced La-Deficient LFM Samples

**Figure 5:**
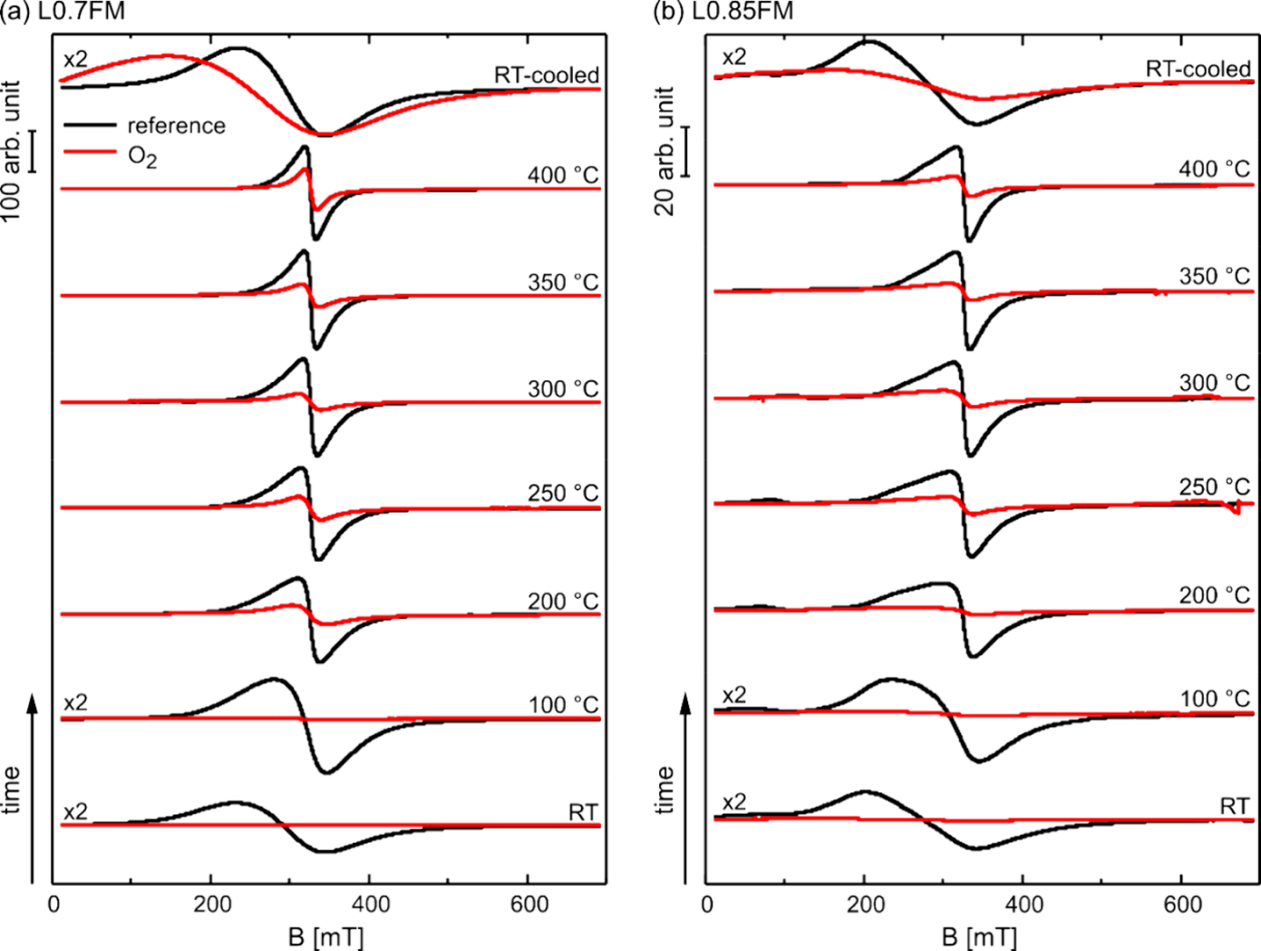
*In situ* cw ESR spectra obtained at different temperatures for reoxidation by heating in an O_2_-containing atmosphere: (a) L0.7FM and (b) L0.85FM, which were previously reduced by heating in a H_2_-containing atmosphere (red traces). The spectra are displayed together with the reference measurements obtained for heating the pristine (i.e., nonreduced) samples in an O_2_-containing atmosphere (black traces).

The two LFM samples reduced by heating in H_2_-containing atmospheres were subsequently heated in an O_2_-containing atmosphere (10 vol % O_2_ in N_2_, as used in the reference measurements) to investigate if the chemical reduction by heating in a H_2_-containing atmosphere can be reversed. The *in situ* ESR spectra obtained during the reoxidation by heating in the O_2_-containing atmosphere are displayed in Figure 5 together with the reference spectra obtained by heating the pristine, i.e., nonreduced, samples heated in a O_2_-containing atmosphere. Upon heating the reduced samples in an O_2_-containing atmosphere, the ESR signals gain in intensity, indicating a reoxidation of the samples and the formation of a ferromagnetic phase. The reoxidation is expected to replenish lattice oxygen, which facilitates increased superexchange and/or Mn^4+^ formation. For both samples, the first indication for an increase in ESR signals is detected already at 100 °C (see also Figure S10) coinciding with the onset of the O_2_ uptake reported in a previous study for samples reduced by heating to 700 °C in a H_2_-containing atmosphere.^19^ Thus, the onset temperature for changes in the ESR signals due to reoxidation is similar for the two samples and results in signals compatible with the high field (*g*_eff_ ≈ 2) signal discussed above for both samples, which were found to disappear at comparable temperatures during reduction. However, despite comparable O_2_ uptake,^19^ the initial intensity increase in ESR signal at 100 °C is more pronounced for the L0.85FM sample (see also Figure S10).

Upon heating the reduced samples in an O_2_-containing atmosphere, the previous study reported the majority of the O_2_ uptake for both samples to occur for temperatures between 250 and 400 °C.^19^ However, the ESR signals remain remarkably stable in this temperature range except for an increase at 400 °C observed for L0.7FM (see also Figure S7). Importantly, both samples exhibit smaller signals as compared to the reference measurements at 400 °C. A possible reason for the observed differences may be an incomplete oxygen replenishment. Uptake studies indeed show a higher hydrogen uptake during reduction than oxygen uptake during reoxidation, but only the L0.85FM sample has a significant oxygen deficiency of about 50% as compared to the pristine sample, whereas the deficiency of the L0.7FM sample was found to be around 10% (when comparing H_2_ and O_2_ uptake for heating up to 400 °C).^19^ Considering the cw ESR signal intensity at 400 °C compared to the expected oxygen replenishment, the signal intensity is not simply linearly correlated with the amount of replenished oxygen. While the qualitative changes of the reoxidized samples during heating are similar, significant differences between the two samples become immediately obvious, if the spectra observed after cooling the sample back down to RT (Figure 5, top traces) are compared. In the case of the L0.7FM sample, the signal at RT (red trace) is shifted to lower fields and has a comparable signal amplitude as the reference; however, its line width is significantly increased. This corresponds to a significant increase in signal intensity, which could be estimated from a double integration of both spectra to amount to an increase in at least 50%, as compared to the reference. In contrast, the signal of L0.85FM, which is also significantly broadened, has a much smaller signal amplitude. Estimation of the signal intensity reveals a reduction by about 25% as compared to the reference spectrum indicating a smaller magnetization of this sample after reoxidation.

**Figure 6:**
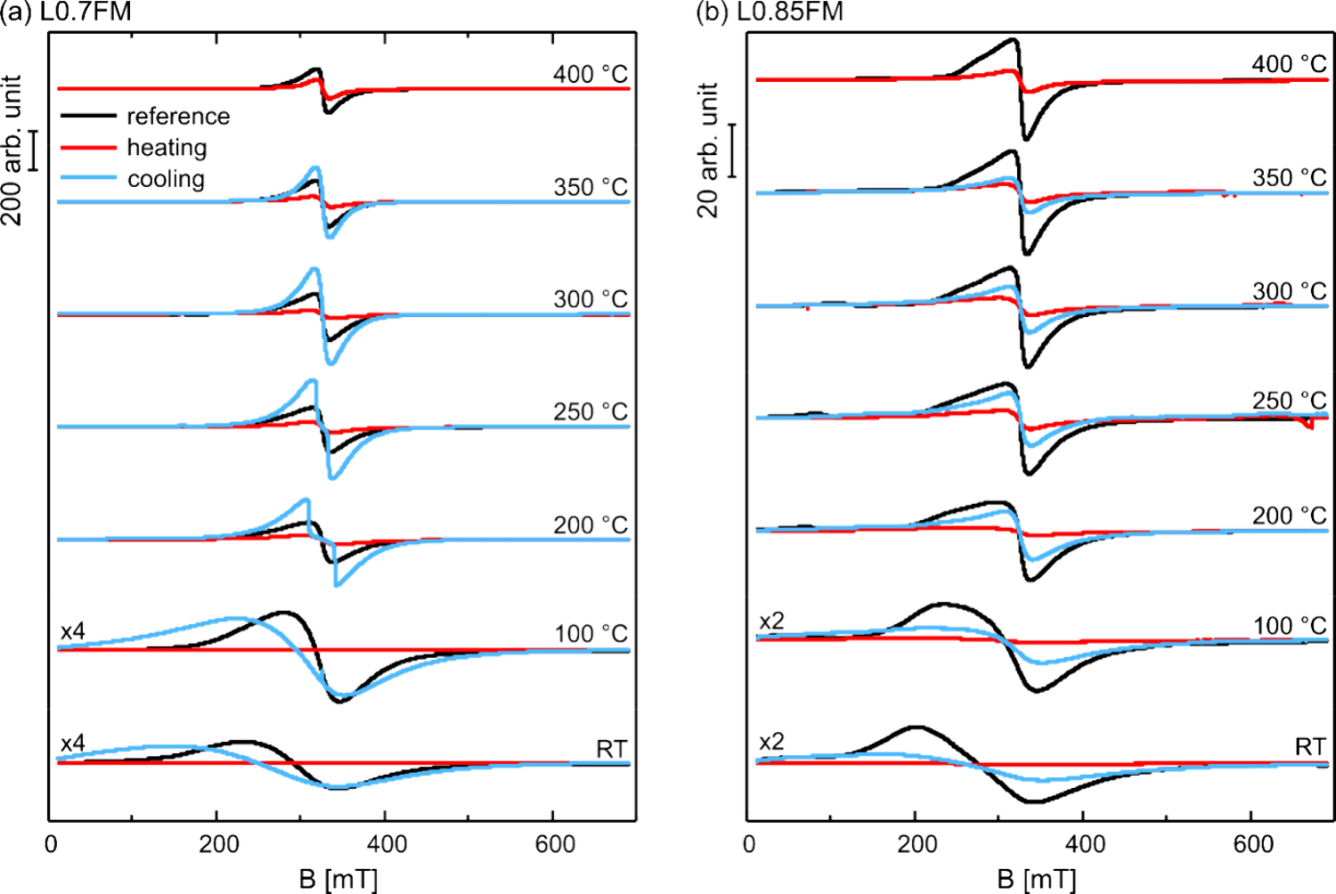
*In situ* cw ESR spectra obtained at different temperatures during reoxidation by heating in an O_2_-containing atmosphere of (a) L0.7FM and (b) L0.85FM, which were previously reduced by heating in a H_2_-containing atmosphere. Next to the spectra obtained during the heating of the reoxidation (red traces, also shown in Figure 5), also the spectra obtained during the subsequent cooling steps of the reoxidation are shown (blue traces). The spectra are displayed together with the reference measurements obtained for heating the pristine (i.e., nonreduced) samples in an O_2_-containing atmosphere.

The significant change of the ESR signal observed between 400 °C and RT renders a more detailed analysis of the evolution of the spectral changes as a function of the temperature interesting. The corresponding spectra are shown in Figure 6. Upon cooling from 400 to 350 °C, the signal intensity increases significantly in both cases (see also Figure S7). While the signal intensity at 350 °C is higher than the one observed at the same temperature during heating for both samples, the L0.7FM signal exhibits a signal that is even larger than the one observed for the reference sample. For the L0.7FM sample, two important conclusions can be drawn at this point: 1. The larger magnetization at 350 °C indicates a significant change of the magnetic properties in the reoxidized sample compared to the reference. 2. The reversal of the intensity ratio of the reoxidized L0.7FM and the corresponding reference sample, when comparing the signals at 400 °C with those at 350 °C, points to a reduction of the Curie temperature for the reoxidized sample, which has to be close to 400 °C to account for the observed effect. Lowering the temperature further first increases the signal intensity of the L0.7FM spectrum without a significant change of the line shape (300 °C, Figure 6a). The latter is, however, altered considerably at 250 and 200 °C, as evidenced by the development of a plateau region in between the extrema of the signal. Such changes in the line shape can be expected for superparamagnetic particles at a temperature where the dynamics of the magnetic moments is slowed down sufficiently that magnetic anisotropies, which may include magnetic interactions between grains of the nanostructured material, alter the line shape of the ESR signal. The effect of magnetic anisotropies becomes even more substantial at lower temperatures, which results in a further broadening of the line shape. The corresponding change in magnetization dynamics also removes the peculiar line shape observed at 200 and 250 °C. Field-dependent magnetization measurements of the reoxidized L0.7FM sample showed a behavior expected for superparamagnetic particles differing, however, from the pristine sample with, e.g., an increased remanence at low temperatures and a higher magnetization at RT. This is in line with the ESR results (see also Figure S11). Please note that the timescales of ESR and magnetization measurements are considerably different. Thus, superparamagnetic behavior is observed in magnetization measurements at lower temperatures than in ESR, which probes the magnetization on a much smaller timescale. *Ex situ* XRD spectra of the reoxidized L0.7FM sample were performed (Figure S3), which indicate that the resulting material contains the same crystalline phase as the initial sample. No other crystalline phases were observed. In particular, no evidence for a MnFe_2_O_4_ spinel phase was found, which was previously reported to form during reoxidation in pure O_2_ around 270 °C for samples more severely reduced by heating to 700 °C in pure H_2_.^19^ Moreover, the lattice constants and the volume of the unit cell for the perovskite phase are only slightly altered, as compared to the pristine samples (see Table 1). Hence, the *ex situ* XRD results do not provide evidence for significant changes in the crystalline perovskite phase after the reoxidation, which could readily explain the observed changes in the magnetic properties.

The behavior of the L0.85FM sample has some resemblance to that of the L0.7FM sample but also significant differences. Also, for the L0.85FM sample, the signal intensity of the ESR spectra of the cooling series is always higher than observed during heating (Figure 6b). However, the signal intensity remains smaller than that of the reference sample, in contrast to observations for the L0.7FM sample. The signal intensity for L0.85FM increases with decreasing temperature, and the line shape exhibits a second line on the low field side of the main signal similar to the one observed during the reduction in hydrogen. The line shape remains similar down to 200 °C. Below that, the signal broadens significantly, resulting in a drop of signal amplitude, which is qualitatively similar to the behavior found for the L0.7FM sample. Qualitatively, such a broadening can be rationalized by reduced magnetization dynamics of the superparamagnetic crystallites due to the impact of magnetic anisotropies on the line shape at lower temperatures. Also, for this sample, *ex situ* XRD has been measured for the reoxidized sample and the results are very comparable to the L0.7FM sample. In particular, no indication of additional phases was found. The diffractogram solely contains peaks that can be assigned to the known perovskite phase, again with only slight changes in lattice parameters, as compared to the pristine sample. Despite the similarities in the XRD signature of both samples after a reduction and reoxidation cycle, the magnetic properties reveal clear changes indicating that irreversible changes of the samples are taking place.

## Discussion

*In situ* ESR measurements were conducted to investigate the reduction and (re)oxidation of ferromagnetic LFM samples with varying degrees of La deficiency. While the ESR spectra of these samples do not exhibit any sharp signals due to isolated paramagnetic species, broad signals are observed, as expected for materials with collective magnetism. These *in situ* cw ESR investigations demonstrated that cw ESR is sensitive not only to defects connected to the La deficiency but also clearly to changes upon reduction or reoxidation. The results from this work and prior work for the investigated samples are summarized in Table 2. While the sample with the higher La deficiency exhibits a more intense ESR signal, reduction in a H_2_-containing atmosphere at elevated temperatures was found to quench the ESR signals (400 °C) for both samples, which is associated with the removal of lattice oxygen. It was shown that reduction of the sample can occur at temperatures above 300 °C in “inert” gas (N_2_), which is, however, significantly less effective than H_2_ in reducing the samples. The L0.85FM sample exhibits a magnetic signal that behaves distinctly different from the main component of the spectrum, as this signal increases in intensity between 200 and 300 °C in the reductive environment, while the main signal remains stable until 300 °C, before both decrease at higher temperatures. Importantly, this second component is observed only for the L0.85FM sample and not present in the more La-deficient sample (L0.7FM). XRD characterization shows only one perovskite phase. While STEM measurements provide no evidence for a second crystalline phase in the pristine samples, the results attested to a heterogeneity demonstrating Fe-enriched and La-depleted regions at the interface between particles, which is in line with previous XPS results also indicating a substantial compositional difference in the surface region as compared to the bulk composition.^19^ This heterogeneity evidenced by STEM may critically affect the magnetization of the samples, but no direct correlation is evident to the heterogeneity probed by cw ESR. The latter is tentatively attributed to differences in the surface composition previously reported for the pristine La-deficient samples.^19^ These results clearly demonstrate the ability of the magnetic properties to serve as a very sensitive probe, which allows to identify heterogeneity in these samples not readily observed in commonly used structural characterization techniques such as XRD or TEM. Importantly, the qualitatively different responses of the two magnetic phases to reducing conditions also imply that the heterogeneity may critically affect the reactivity of the materials.

**Table 2:** Comparison of Results for L0.7FM and L0.85FM Summarizing Results of This Work (Asterisks) and a Prior Study^a^

**sample**	**observation (detection method)**	**L0.7FM**	**L0.85FM**
**pristine**	- LFM pervoskite phase (XRD)^19,^*	+	+
	- ESR signal at g ≈ 2 ((*in situ*) ESR)^19,^*	+	+
	- Fe-enriched and La-depleted regions (STEM)*	+	+
	- 2nd ESR signal *g* > 2 (*in situ* ESR)*	−	+
	- La-enriched surface layer (XPS)^19^	×	+
**reduced**	- *in situ* ESR signal change T ≈ 200 °C*		
	- g ≈ 2	×	×
	- g > 2	−	+
	- *in situ* ESR signal intensity lower*		
	- g ≈ 2, T ≈ 300 °C	+	+
	- g > 2, T ≈ 350 °C	−	+
	- H_2_-uptake^19^		
	- starts at T ≈ 170 °C	+	+
	- rises at T ≈ 300 °C and slows at 350–400 °C	+	+
	- change of ESR signal in N_2_*	>350 °C	≈350 °C
	- formation of Fe^0^ particles		
	- in H_2_ (XRD)^19^	+	+
	- in 10 vol.% H_2_ (ESR)*	×	×
	- MnO, La_2_O_3_ in pure H_2_ at 640 °C (*in situ* XRD)^19^	+	+
**reoxidized**	- ESR signal increase starts at ≈100 °C*	+	+
	- ESR signal higher/lower than reference*		
	- at RT	higher	lower
	- at T = 400 °C	lower	lower
	- higher RT magnetization than reference*	+	
	- O_2_-uptake^19^		
	- starts at ≈100 °C	+	+
	- smaller than prior H_2_-uptake	+	++
	- peculiar ESR line shape in cooling at 200–250 °C*	+	×
	- LFM perovskite phase (XRD)^19,^*	+	+
	- MnFe_2_O_4_ and La_2_O_3_ (XRD)		
	- after reduction in pure H_2_ to 700 °C^19,^	+	+
	- after reduction in 10 vol.% H_2_ ≤ 400 °C*	×	×
**spent**	- LFM perovskite phase (XRD^19^/STEM*)	+	+
	- agglomerated particles (STEM)*	+	+
	- higher remanence and magnetization at 4 K than pristine*	+	+
	- ESR signal shifted and higher line width than pristine*	++	+
	- higher RT magnetization at 5 T than pristine*	+	×
	- Fe-enriched and La-depleted regions (STEM)*	+	×
	- MnFe_2_O_4_ spinel phase (XRD)^19^	+	×
	- coercivity at 4 K higher than pristine (magnetization)*	×	+
	- surface reduction (O 1s, XPS)^19^	(+)	++

a + indicates observation of an effect; ++ indicates a strong effect; a cross mark indicates that the effect is not detected; “−” emphasizes that L0.7FM does not show a second ESR signal at *g* > 2.

The removal of lattice oxygen through H_2_ reduction results in a loss of the ESR signal at a high temperature for both samples. The reoxidation of the reduced sample allows to re-establish ESR signals for both of the samples, which are, however, significantly altered as compared to the pristine sample. More importantly, significant differences are observed for the two samples. While the cw ESR signal intensity remains below that of the pristine sample for reoxidized L0.85FM, which is consistent with incomplete (50%) reoxidation reported previously under these conditions,^19^ the cw ESR intensity of L0.7FM after reoxidation even exceeds that of the pristine sample, even though the uptake studies provide no evidence for an overoxidation of the sample.^19^ Hence, the higher ESR signal intensity of reoxidized L0.7FM as compared to the pristine sample points to a more complex change in magnetic properties. First, the increase in signal intensity already at 350 °C as compared to the reference clearly points to a reduced Curie temperature, which has to be close to 400 °C for the reoxidized sample. Furthermore, the complex line shape observed between 250 and 200 °C suggests a heterogeneity of the effective magnetic anisotropy in the sample giving rise to this behavior. These results clearly attest to significant differences in the magnetic properties of reoxidized and pristine samples, while no significant changes in the phase contribution could be observed by XRD. Reoxidation induces smaller deviations in the magnetic properties of L0.85FM than for the L0.7FM sample, but the changes compared to the pristine sample are still significant, as seen, e.g., by the strong increase in the line width in the RT spectra. A possible origin for the observed effects that are particularly obvious for the L0.7FM signal could be formation of a second magnetic phase, as it is observed for the pristine L0.85FM sample. While *ex situ* XRD did not give evidence for a second crystalline phase in the reoxidized sample, previous investigations reported an overall lower structural stability for the L0.7FM sample than for the L0.85FM sample.^19^ A lower structural stability during reduction and reoxidation may cause a compositional redistribution and also agglomeration of the particles in the sample. Next to changes in the composition or distribution, also agglomeration, i.e., changes in the particle size or the interface between different particles, will affect the ESR spectra and overall magnetization in the sample, as such changes alter the effective magnetic anisotropies by changing the volume of the superparamagnetic phases or magnetic coupling between adjacent particles.

To this end, it is interesting to note that STEM investigations of these two samples show the presence of agglomerated nanocrystalline particles after samples have been used as catalysts in the reaction of NO with CO (“spent catalyst”, see Figures S12 and S13). While the material still exhibits crystallites with a perovskite structure also found for the pristine sample, the treatment in the reactive gas phase alters the association of the crystallites. For the spent L0.7FM catalyst, there are still clear signs for a heterogeneous distribution of La and Fe. However, we were not able to see a similar trend in the spent L0.85FM catalyst.

**Figure 7:**
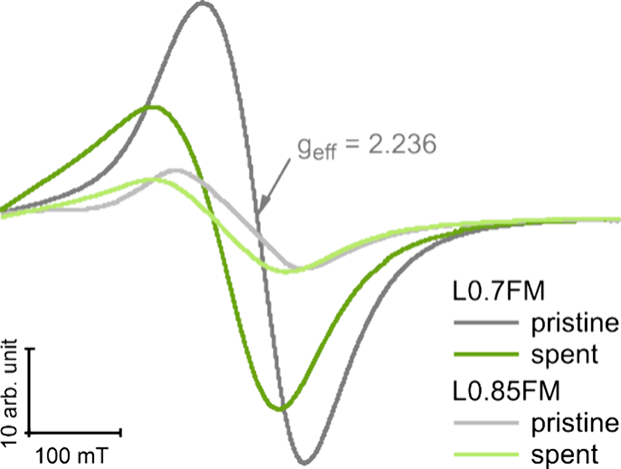
*Ex situ* ESR measurements of L0.7FM (dark traces) and L0.85FM (light traces) before (i.e., pristine, gray traces) and after the reaction with NO and CO (green traces).

*Ex situ* cw ESR measurements of the spent catalysts were exemplarily conducted to test if also the cw ESR signals are affected after the catalytic reaction, which are displayed together with the spectra of the pristine samples for comparison (Figure 7). After the reaction with NO and CO, clear changes in the cw ESR spectra are observed, demonstrating that also the catalytic reaction alters the magnetic properties in the LFM samples, in qualitative agreement with expectations from the measurements during reduction and reoxidation. The signals observed after the catalytic reaction show a shift to lower magnetic fields as well as an asymmetric broadening of the line on the low field side. Both of these effects are consistent with an increase in the effective magnetic anisotropy. In agreement with the notion of changes in the magnetic phases in the spent samples, field-dependent magnetization measurements at 4 K show an increased remanence and magnetization at 5 T (Figure S14). While the changes in the ESR signals are qualitatively similar for both samples, the shift in *g*_eff_ and broadening of the line width are significantly more pronounced for L0.7FM than for L0.85FM, in qualitative agreement with the results of the redox cycle. Also, field-dependent magnetization measurements show differences for the two spent samples, such as an increased coercivity at 4 K for L0.85FM and a slightly increased RT magnetization at 5 T for L0.7FM (Figure S14). The more pronounced changes in ESR for L0.7FM are consistent with the previous study reporting L0.7FM to be more reduced and structurally less stable in the reaction with CO and NO than L0.85FM.^19^ It is interesting to note that the cw ESR spectra of L0.7FM are more strongly affected by the chemical reaction with NO and CO or H_2_ and subsequently O_2_ than those of L0.85FM, while purely thermal reduction by heating in pure N_2_ induces more pronounced changes in the L0.85FM spectra suggesting a complex redox behavior of the samples with varying degrees in La deficiency.

These cw ESR results clearly demonstrate characteristic signal changes for varying degrees of La deficiency and redox states of the perovskite material, attesting to the sensitivity of the method for such systems. They also provide evidence for heterogeneity in the samples not detected by XRD and different from that evidenced by STEM yielding, thus, additional information on such complex materials. Furthermore, first evidence is provided that the observations obtained for reduction and reoxidation of the samples with different La deficiency, i.e., the distinct heterogeneity in magnetic phases and their diverse redox response to different atmospheres, may enhance the understanding of their catalytic activity in reactions involving changes in the redox state of the catalysts, such as the reduction of NO with CO. Thereby, the *in situ* cw ESR investigations conducted in different atmospheres may also aid in a rational approach for future improvements of such defect-rich perovskite materials for catalytic but also other applications involving reaction or processing steps at high temperatures.

## Conclusions

In this work, temperature-dependent *in situ* cw ESR measurements were conducted to investigate the changes in the magnetic phases of La-deficient LFM samples. While heating to high temperatures in pure N_2_ resulted in thermally induced reduction of the defect-rich perovskite samples, this process can be suppressed by heating in an O_2_-containing atmosphere preventing changes in the magnetic properties as probed by ESR spectroscopy. This allows to use the corresponding temperature-dependent spectra as a reference to identify changes in the magnetic properties induced by changes in the chemical composition of the samples. Heating in a H_2_-containing atmosphere facilitates reduction of the samples, which occurs at lower temperatures and results in a more reduced sample as compared to a thermal reduction in pure N_2_. While ESR signals associated with La deficiency enhance the ESR intensity, reduction at high temperatures lowers the signal intensity strongly, which can be understood by removal of oxygen from the perovskite materials. Yet, for a second magnetic phase present in the sample with a lower La deficiency (L0.85FM), reduction in a H_2_-containing atmosphere results in an intensity increase in the ESR signal for intermediate temperatures (200–300 °C) indicating a qualitatively different impact of reduction on the magnetic properties and importantly also a response to the reducing conditions at lower temperatures. Thus, it is expected that this magnetic phase can also affect the reactivity of L0.85FM in redox reactions at low temperatures. As *ex situ* XRD measurements only evidenced one crystalline phase, the observation of heterogeneity by cw ESR provides important additional insight into the structural properties, which might also be related to the differences in the catalytic properties observed for L0.85FM as compared to L0.7FM. While STEM measurements do not provide evidence for a second phase, which could be associated with heterogeneity observed in ESR, these results provide evidence for heterogeneity in composition within the crystallites. As site occupation in perovskite materials is intimately linked to magnetic properties, the STEM results provide experimental evidence for the possibility that magnetic coupling between the paramagnetic ions within such lattices can depend on the location within a crystallite. Depending on the initial composition and its evolution during reduction, it is well-conceivable that the magnetic properties are altered by such heterogeneities, which contribute at least in part to the observed differences of the two samples. The heterogeneity may also be connected with the stability of the samples upon reduction and reoxidation, as the cw ESR spectra show larger deviations from the pristine sample for the sample with higher La deficiency without the second magnetic phase. The sample with the higher La deficiency exhibits stronger changes in the magnetization after reoxidation, which may also be connected to agglomeration, as observed by STEM measurements of spent samples, i.e., after the catalytic reaction. The low-temperature reactivity under reducing conditions and the difference in structural stability in redox cycles depending on the La deficiency and the connected heterogeneity of the perovskite are both expected to affect the catalytic reactivity in redox reactions, such as the reaction of CO and NO. The cw ESR results of the spent samples are consistent with particle agglomeration and yield distinct changes in the magnetic phases for the samples with different reactivity, indicating that the different magnetic phases may also be connected to the differences in reactivity of the La-deficient LFM samples in this reaction.

## Data Availability

Raw and meta data are available under doi 10.5281/zenodo.14918212

## Supporting Information Available

Magnetization measurements as a function of temperature and magnetic field (4 and 300 K) of L0.7FM and L0.85FM of pristine and spent samples as well as for reoxidized L0.7FM; XRD measurements of L0.7FM and L0.85FM for pristine samples as well as for samples after heating in pure N_2_ or an O_2_-containing atmosphere and for reoxidized samples; cw ESR measurements of L0.7FM and L0.85FM for different temperatures during heating and cooling in pure N_2_ and in an O_2_-containing atmosphere, obtained at 430 °C for heating in pure O_2_ and an O_2_-containing atmosphere, taken at RT before and after preoxidation, obtained during heating for reoxidation at 100 °C, and cw ESR measurements obtained at 300 °C for heating in a H_2_-containing atmosphere for L0.85FM; (double) integrated intensity of cw ESR signals during reduction in a H_2_-containing atmosphere and reoxidation in an O_2_-containing atmosphere; STEM measurements for pristine L0.85FM as well as for spent L0.7FM and spent L0.85FM (PDF)
