# Supplementary material for: In Situ Cw ESR Study on Redox Behavior and Phase Heterogeneity in A-Site-Deficient Lanthanum Iron Manganite Perovskite Catalysts
Source: J Phys Chem C Nanomater Interfaces. 2025 Apr 7;129(15):7413–26. doi: 10.1021/acs.jpcc.4c08262 (PMC12010419; doi:10.1021/acs.jpcc.4c08262)
Supplement: Supplementary file 1 — jp4c08262_si_001.pdf [file jp4c08262_si_001.pdf]

**Supporting Information**

# In-situ cw ESR Study on Redox Behavior and Phase Heterogeneity in A-Site Deficient Lanthanum Iron Manganite Perovskite Catalysts

Zohreh Asadi,[a] Asghar Mohammadi,[b] Thomas Ferdinand Winterstein,[b] Ralf Feyerherm,[c] Roham Talei,[d] Nicolas Bonmassar,[d] Wiebke Riedel,[a] Simon Penner,[b] Thomas Risse[a,*]

[a] *Institut für Chemie, Freie Universität Berlin, Arnimallee 22, 14195, Berlin, Germany*

[b] *Institute of Physical Chemistry, University of Innsbruck, Innrain 52c, A-6020, Innsbruck, Austria*

[c] *Institute Quantum Phenomena in Novel Materials, Helmholtz-Zentrum Berlin für Materialien und Energie GmbH, Hahn-Meitner-Platz 1,14109 Berlin, Germany*

[d] *Institute for Materials Science, University of Stuttgart, Heisenbergstr. 3, 70569 Stuttgart, Germany*

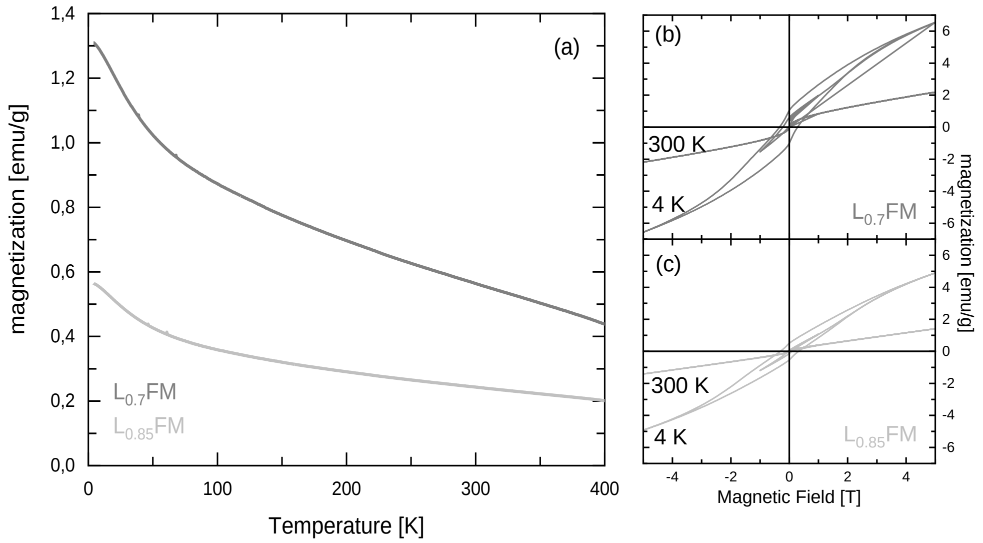


**Figure S1** Magnetization measurements of pristine L0.7FM (dark grey traces) and L0.85FM (light grey traces) as a function of temperature (a) and function of magnetic field at 4 K and 300 K (b,c). Temperature and magnetic field dependent magnetization measurements have been carried out on a Quantum Design Physical Properties Measurement System (PPMS). Small amounts of sample (8-10 mg) were filled into plastic capsules and mounted to brass sample holders. For each sample, the temperature dependent magnetization between 2 K and 400 K has been measured after field cooling from 300 K to 2 K at an external field of 0.5 T. At 4 K and 300 K, full hysteresis curves have been measured up to 5 T.

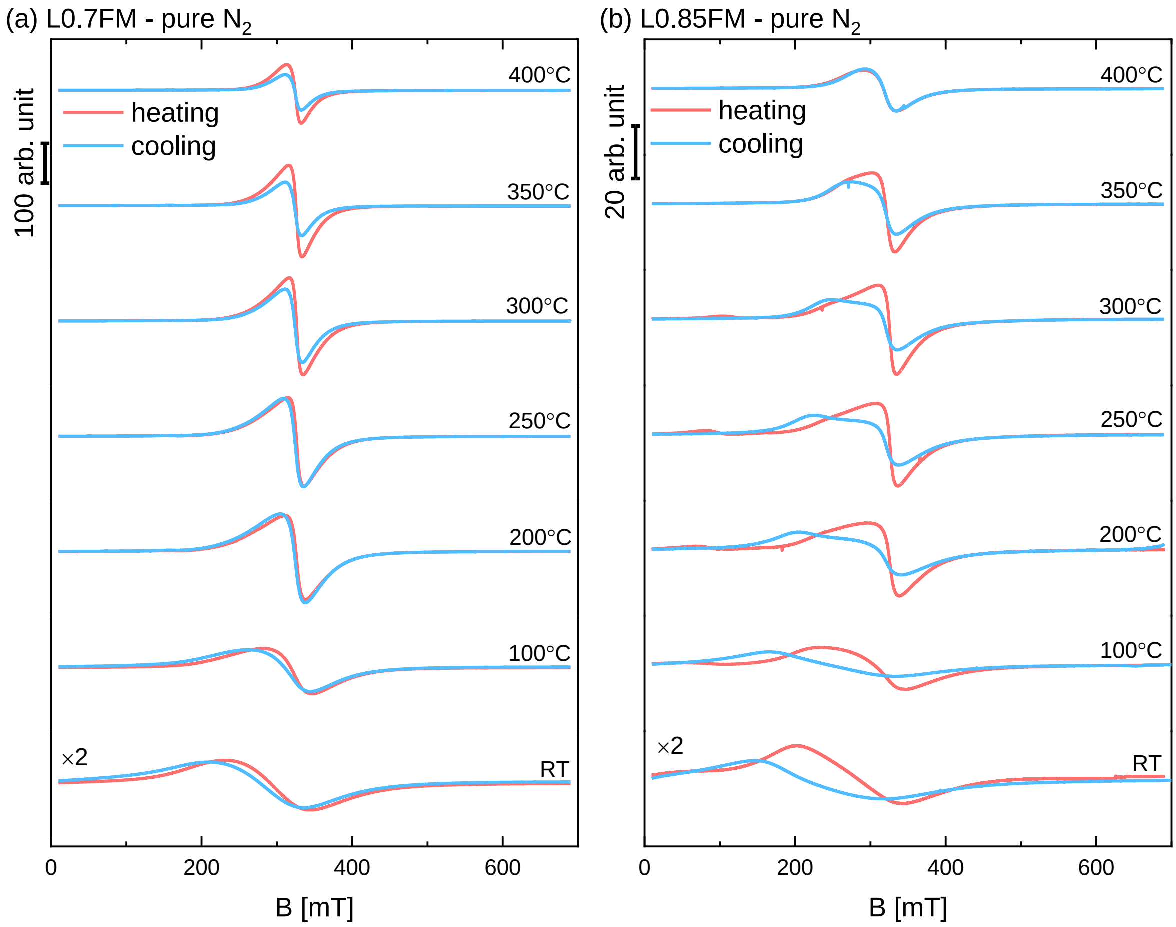


**Figure S2** *In-situ* cw EPR spectra of (a) L0.7FM and (b) L0.85FM obtained at different temperatures for heating (red traces) and cooling (blue traces) in pure $N_2$. Clearly, the spectra taken at the same temperature differ for the heating and cooling steps indicating changes of the samples due to heating in pure $N_2$.

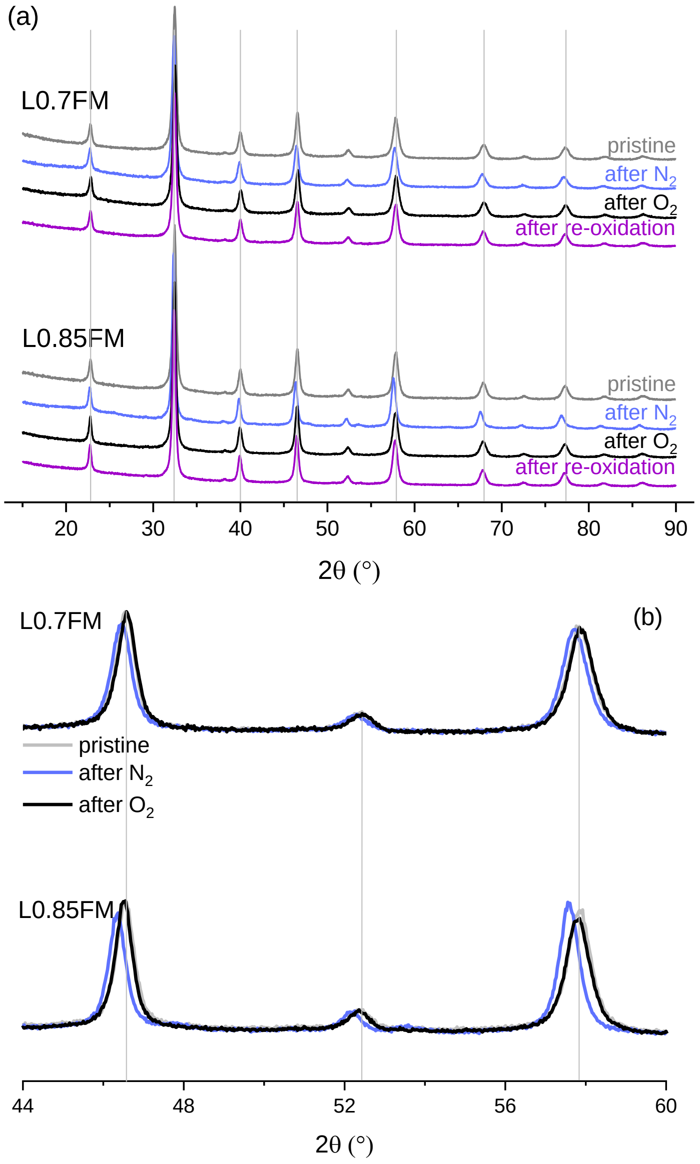


**Figure S3** *Ex-situ* XRD results of L0.7FM and L0.85FM samples measured for pristine samples and after heating in pure $N_2$ and in $O_2$-containing atmosphere. Additionally, results for the pre-oxidized samples which were reduced by heating in $H_2$-containing atmosphere and subsequently re-oxidized by heating in $O_2$-containing atmosphere. While (a) shows the diffraction pattern in the entire measurement range, in (b) a smaller range is displayed to show the shifts in diffraction reflexes after heating in pure $N_2$. The signal intensity is normalized for each sample to the maximum amplitude of the signal at $2\theta \approx 32°$.

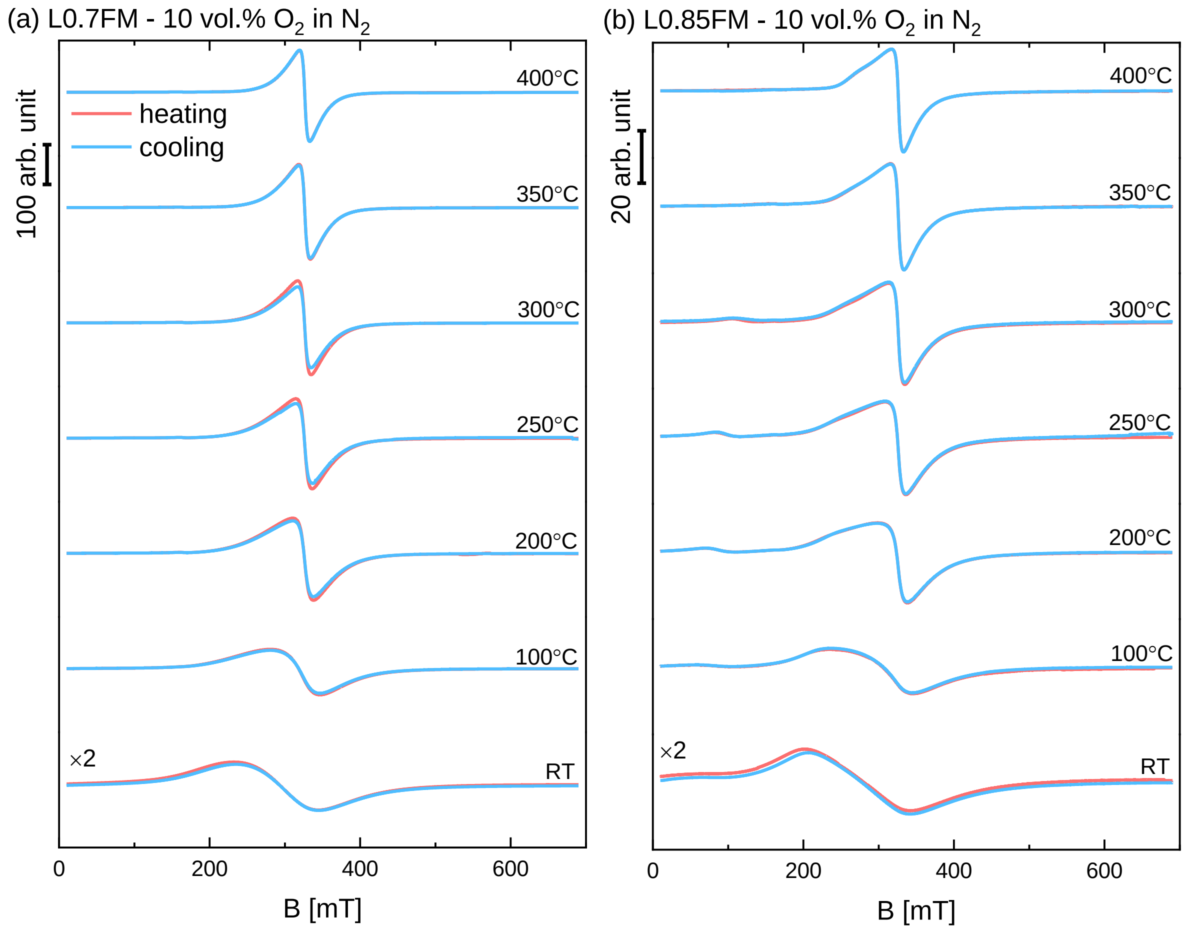


**Figure S4** *In-situ* cw EPR spectra of (a) L0.7FM and (b) L0.85FM obtained at different temperatures for heating (red traces) and cooling (blue traces) in oxygen-containing atmosphere (10 vol.% $O_2$ in $N_2$). In contrast to the measurements obtained in pure $N_2$ (see Figure S2), the spectra taken at the same temperature are essentially identical for the heating and cooling steps. Therefore, this set of measurements was used as reference.

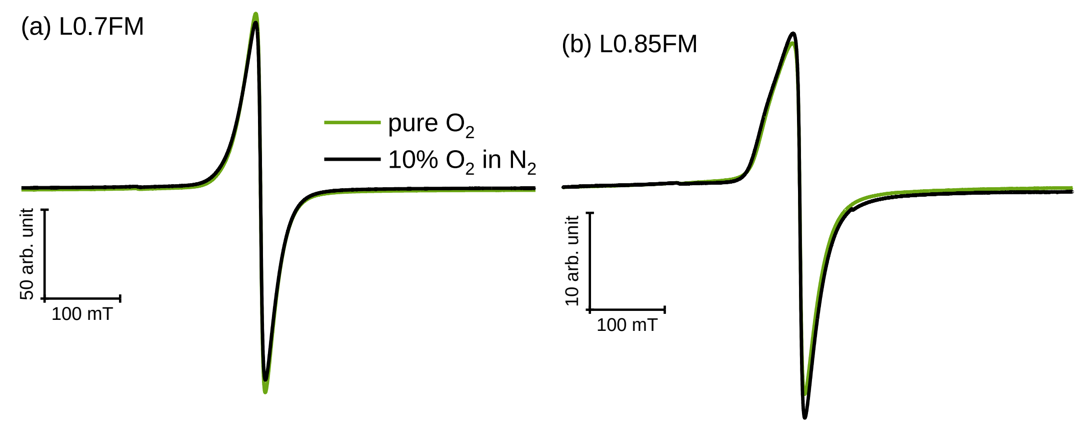


**Figure S5** *In-situ* cw EPR spectra obtained at 430 °C in oxygen-containing atmosphere (10 vol.% $O_2$ in $N_2$) and in pure $O_2$ for (a) L0.7FM and (b) L0.85FM.

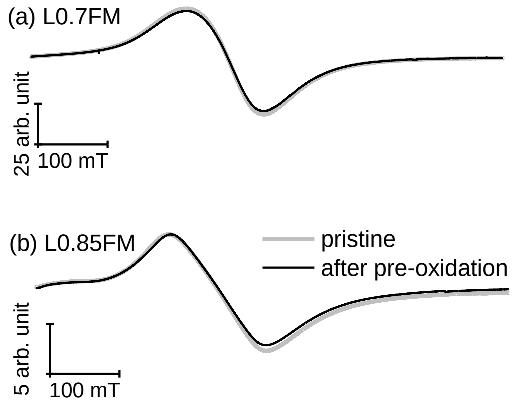


**Figure S6** Cw EPR spectra of (a) L0.7FM and L0.85FM obtained at RT before (i.e. pristine, grey traces) and after pre-oxidation, i.e. heating in pure $O_2$ to 430 °C (black traces).

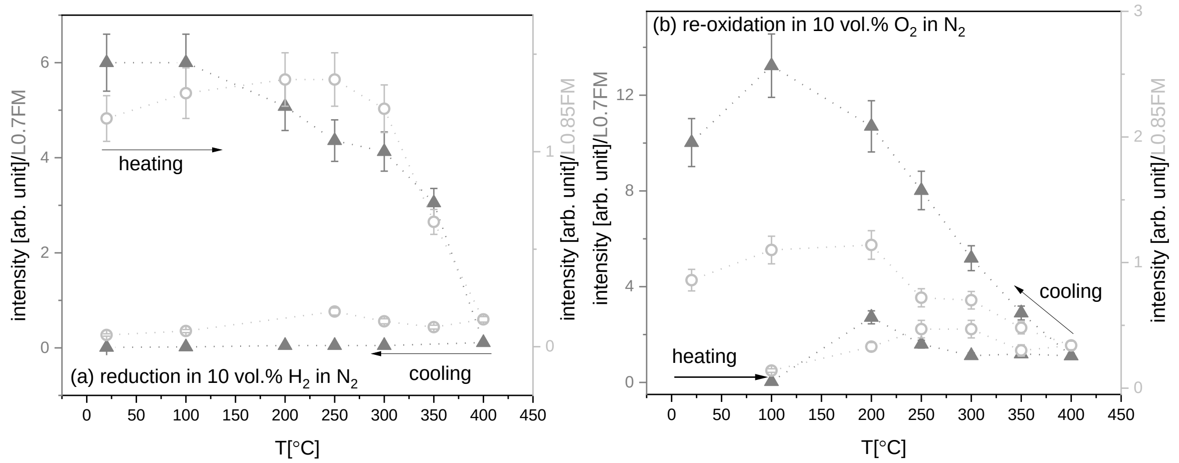


**Figure S7** (Double-)integrated intensity of *in-situ* cw EPR measurements for L0.7FM (filled triangles) and L0.85FM (open circles) for (a) heating and cooling in $H_2$-containing atmosphere as well as for (b) subsequent heating and cooling in $O_2$-containing atmosphere.

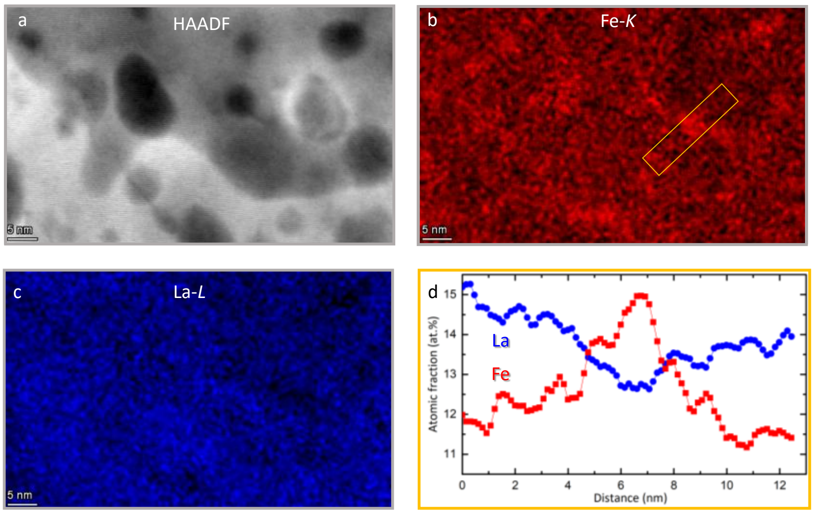


**Figure S8** Nanoscale morphological and quantitative chemical analysis of $La_{0.85}Fe_{0.7}Mn_{0.3}O_3$ using HAADF and EDX mapping. (**a**) HAADF overview image shows agglomerated perovskite nanoparticles. (**b**), (**c**) Elemental mappings of Fe-*K* (red), and La-*L* (blue) peaks. (**d**) Elemental line profile highlighting the atomic fraction integrated at the position of the orange rectangle shown in panel **b**.

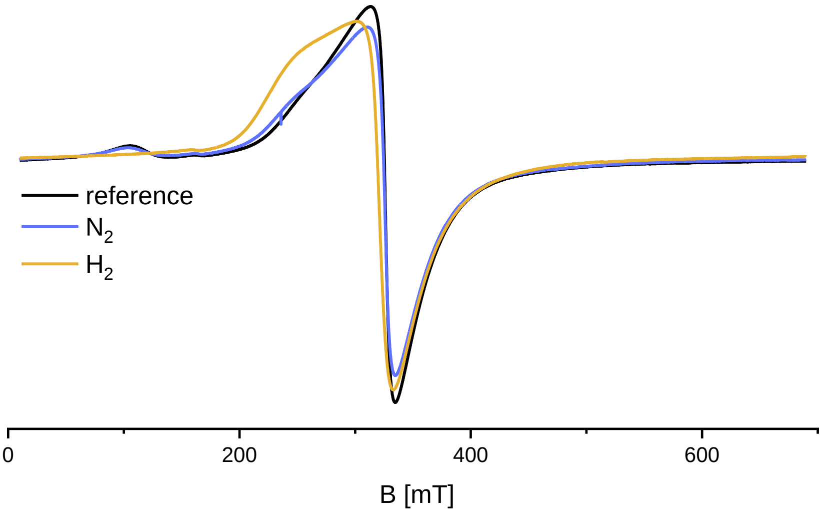


**Figure S9** *In-situ* cw ESR measurements of L0.85FM obtained at 300 °C heating in $H_2$ containing atmosphere (yellow traces) and in pure $N_2$ (blue traces) displayed together with the reference measurements obtained by heating in $O_2$-containing atmosphere (black traces). The spectra which are also shown in Figure 3 are displayed larger to show that also for heating in pure $N_2$ the shoulder at lower magnetic fields gains in intensity, similar to heating in $H_2$-containing atmosphere, even though the effect is less pronounced.

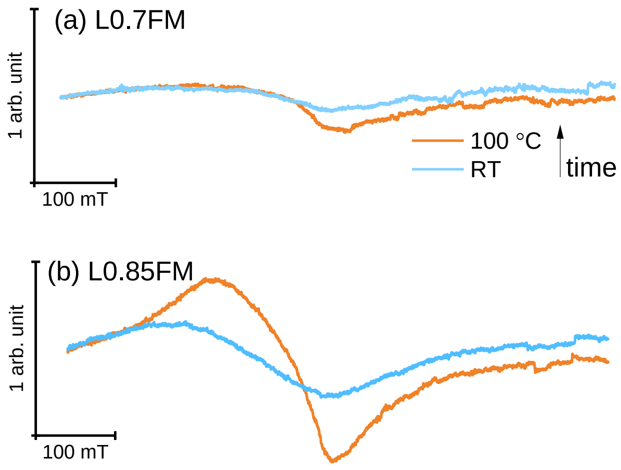


**Figure S10** *In-situ* cw EPR measurements at RT (blue traces) and 100 °C (red traces) for (a) L0.7FM and (b) L0.85FM for re-oxidation by heating in $O_2$-containing atmosphere for the samples which were previously reduced by heating in $H_2$-containing atmosphere.

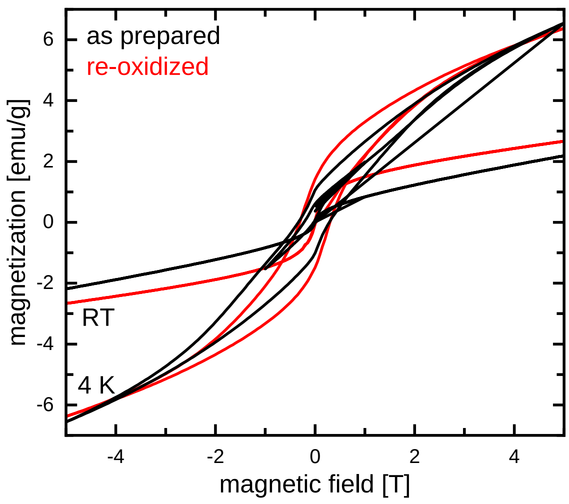


**Figure S11** Field dependent magnetization measurements of re-oxidized L0.7FM conducted at room temperature (RT) and 4 K. For comparison, also results for the as prepared L0.7FM are displayed. At RT, a higher magnetization is found for the re-oxidized sample compared to as prepared L0.7FM. In contrast, at low temperature (4 K), the magnetization is similar. This is consistent with a change in magnetic transition temperature in (at least parts of) the re-oxidized sample.

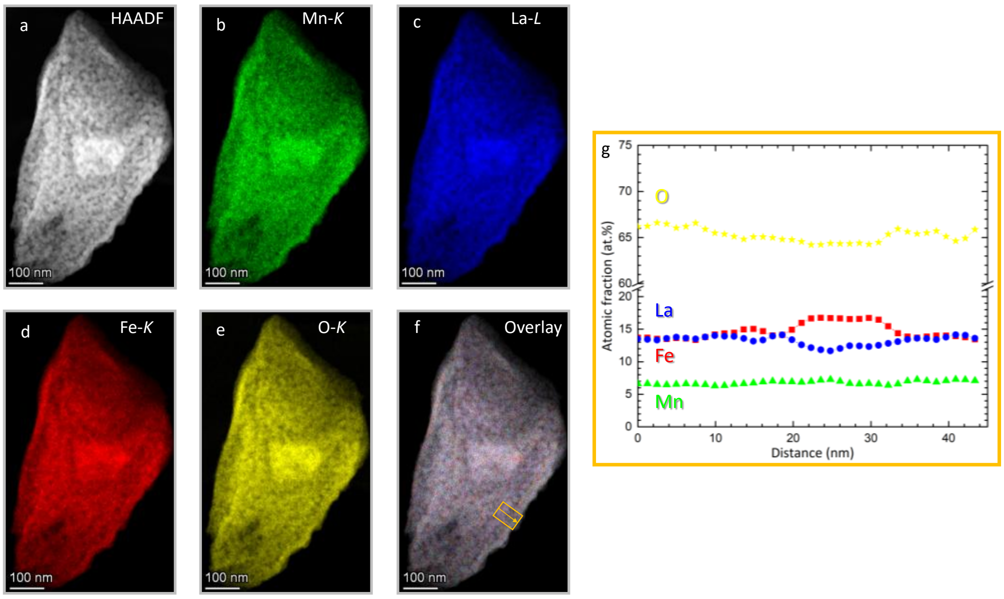


**Figure S12** Nanoscale morphological and quantitative chemical analysis of spent $La_{0.7}Fe_{0.7}Mn_{0.3}O_3$ using HAADF and EDX mapping. (**a**) HAADF overview image shows agglomerated perovskite nanoparticles. (**b**), (**c**), (**d**), (**e**) Elemental mappings of Mn-*K* (green), La-*L* (blue), Fe-*K* (red), and O-*K* (yellow). (**f**) Overlay of La, Mn and Fe signals highlighting the strongly agglomerated nanoparticles with a heterogenous distribution with respect to La and Fe. (**g**) Elemental line profile highlighting the atomic fraction integrated at the position of the orange rectangle shown in panel **f**.

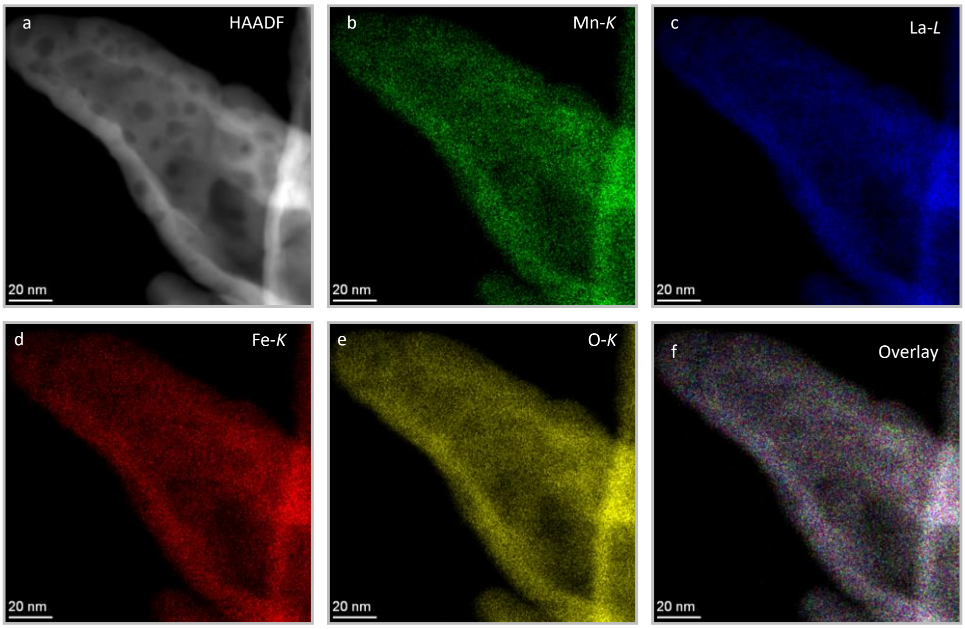


**Figure S13** Nanoscale morphological and quantitative chemical analysis of spent $La_{0.85}Fe_{0.7}Mn_{0.3}O_3$ using HAADF and EDX mapping. (**a**) HAADF overview image shows agglomerated perovskite nanoparticles. (**b**), (**c**), (**d**), (**e**) Elemental mappings of Mn-*K* (green), La-*L* (blue), Fe-*K* (red), and O-*K* (yellow). (**f**) Overlay of La, Mn and Fe signals with no observable signs for heterogeneity.

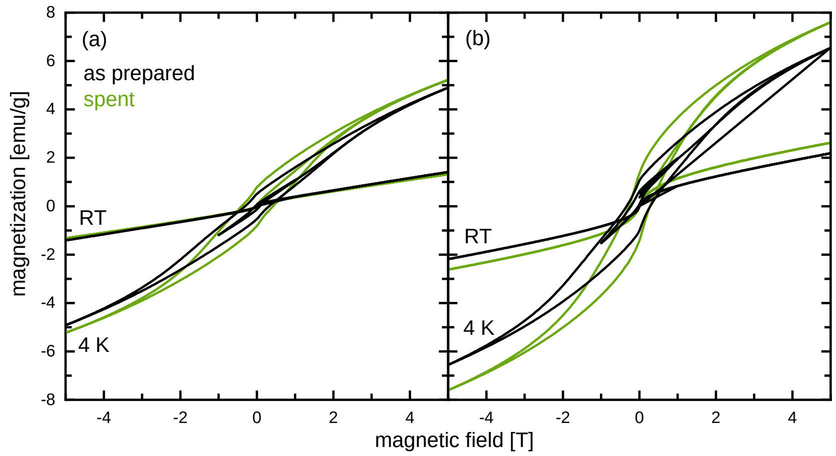


**Figure S14** Field dependent magnetization measurements of the spent (a) L0.85FM and (b) L0.7FM samples measured at RT and 4 K. For comparison, the results for the as prepared samples are also shown. Both samples exhibit changes after reaction, but the changes for spent L0.85FM are rather small (especially at RT), while more notable changes are detected for L0.7FM between the spent and the as prepared sample.
